# Multi-dimensional analysis of B cells reveals the expansion of memory and regulatory B-cell clusters in humans living in rural tropical areas

**DOI:** 10.1093/cei/uxae074

**Published:** 2024-08-12

**Authors:** Mathilde A M Chayé, Oscar R J van Hengel, Astrid L Voskamp, Arifa Ozir-Fazalalikhan, Marion H König, Koen A Stam, Mikhael D Manurung, Yoanne D Mouwenda, Yvonne A Aryeetey, Agnes Kurniawan, Yvonne C M Kruize, Erliyani Sartono, Anne-Marie Buisman, Maria Yazdanbakhsh, Tamar Tak, Hermelijn H Smits

**Affiliations:** Leiden University Center for Infectious Diseases (LUCID), LUMC, Leiden, The Netherlands; Leiden University Center for Infectious Diseases (LUCID), LUMC, Leiden, The Netherlands; Leiden University Center for Infectious Diseases (LUCID), LUMC, Leiden, The Netherlands; Leiden University Center for Infectious Diseases (LUCID), LUMC, Leiden, The Netherlands; Leiden University Center for Infectious Diseases (LUCID), LUMC, Leiden, The Netherlands; Leiden University Center for Infectious Diseases (LUCID), LUMC, Leiden, The Netherlands; Leiden University Center for Infectious Diseases (LUCID), LUMC, Leiden, The Netherlands; Leiden University Center for Infectious Diseases (LUCID), LUMC, Leiden, The Netherlands; Centre de Recherches Médicales de Lambaréné (CERMEL), Lambaréné, Gabon; Parasitology Department, Noguchi Memorial Institute for Medical Research, University of Ghana, Accra, Ghana; Department of Parasitology, Universitas Indonesia, Jakarta, Indonesia; Leiden University Center for Infectious Diseases (LUCID), LUMC, Leiden, The Netherlands; Leiden University Center for Infectious Diseases (LUCID), LUMC, Leiden, The Netherlands; Laboratory for Immunology of Infectious Diseases and Vaccines, Center for Infectious Diseases Control, National Institute for Public Health and The Environment, Bilthoven, The Netherlands; Leiden University Center for Infectious Diseases (LUCID), LUMC, Leiden, The Netherlands; Leiden University Center for Infectious Diseases (LUCID), LUMC, Leiden, The Netherlands; Leiden University Center for Infectious Diseases (LUCID), LUMC, Leiden, The Netherlands

**Keywords:** B cells, host–pathogen interactions, human, parasitic–helminth, proteomics

## Abstract

B-cells play a critical role in the formation of immune responses against pathogens by acting as antigen-presenting cells, by modulating immune responses, and by generating immune memory and antibody responses. Here, we studied B-cell subset distributions between regions with higher and lower microbial exposure, i.e. by comparing peripheral blood B-cells from people living in Indonesia or Ghana to those from healthy Dutch residents using a 36-marker mass cytometry panel. By applying an unbiased multidimensional approach, we observed differences in the balance between the naïve and memory compartments, with higher CD11c^+^ and double negative (DN-IgD^neg^CD27^neg^) memory (M)B-cells in individuals from rural tropical areas, and conversely lower naïve B-cells compared to residents from an area with less pathogen exposure. Furthermore, characterization of total B-cell populations, CD11c^+^, DN, and Breg cells showed the emergence of specific memory clusters in individuals living in rural tropical areas. Some of these differences were more pronounced in children compared to adults and suggest that a higher microbial exposure accelerates memory B-cell formation, which “normalizes” with age.

## Introduction

B-cells are highly versatile and critical in the formation of immune responses against pathogens. B-cells act as antigen-presenting cells, modulate T-cell differentiation and survival, produce pro- or anti-inflammatory cytokines such as IL-2, TNF-α, IL-10, and/or TGF-β [1, 2] and generate immune memory and antibody responses [3].

After maturation and selection of non-autoreactive B-cell receptors (BCRs) in the bone marrow, naïve IgM^+^ B-cells circulate through the body. Upon BCR ligation and migration to secondary lymphoid tissues, B-cells either enter the extrafollicular pathway or the germinal center (GC) pathway. The extrafollicular pathway leads to rapid expansion and differentiation into plasmablasts (PB) and short-lived plasma cells (PC), providing antibodies fast. The GC pathway is slower but generates class-switched antibodies with higher affinity [4–6] and long-lived memory B (MB)-cells, that recirculate through the body, residing in the bone marrow and/or secondary lymphoid organs [7, 8]. Upon re-infection, MB-cells proliferate and differentiate into high-affinity antibody-producing PC [9]. MB-cells consist of different populations, including conventional MB-cells (CD27^+^) and non-conventional MB-cells (CD27^−^). Non-conventional MB-cells contain both atypical MB-cells (defined as CD21^−^CD27^−^ or CD27^−^CD11c^+^), and double negative (DN) MB-cells (IgD^−^CD27^−^), composed of DN type 1 (DN1) and CD11c^+^ DN type 2 (DN2) [10]. Non-conventional MB-cells are present under steady-state conditions, but expand during inflammation and by repeated antigen exposure, such as during infections [11–13], autoimmune diseases [14–18] or immunodeficiencies [19] and therefore are proposed as exhausted or autoreactive B-cells, but their exact role is still subject of research.

B-cells are also important in controlling inflammatory responses to auto-antigens, allergens, or following pathogen clearance, as part of the regulatory immune network; those are referred to as regulatory B (Breg)-cells. Breg cells are heterogenous and various subsets appeared functionally impaired in patients with auto-immunity, allergies, or during graft rejection, while conditions such as gut microbiota, pregnancy, allergen immunotherapy, and chronic infections seem to increase their numbers and/or activity [20–28]. They produce anti-inflammatory cytokines like IL-10, TGF-β, and IL-35, and express inhibitory receptors such as CD1d, PD-L1, PD-1, and FasL, thereby reducing monocyte activation and tissue infiltration and dampening effector Th-cell responses [29–31]. Furthermore, they induce regulatory T (Treg) cells and/or alternatively activated macrophages [29–31].

Constant and/or repeated microbial exposure shapes the immune system and affects its response to pathogens [32–35]. This is more prevalent in rural tropical areas, such as South/South-East Asia, Latin America, the Caribbeans, and Sub-Saharan Africa [36–38], where many parasites like soil-transmitted helminths [39], schistosomes [40] or malaria *Plasmodium falciparum* [41] are endemic. B-cell subset proportions seem dynamic during infection, as reported for various pathogens (e.g. malaria, tuberculosis, chronic helminths, or hepatitis C virus infections) in people living in tropical areas with high pathogen exposure, such as Africa or South-East Asia [42–46].

In this study, we aimed to characterize how B-cell populations are impacted by continuous and substantial pathogen exposition using a 36-marker mass cytometry panel. By applying an unbiased multidimensional approach, we studied B-cell subset differences between regions with higher and lower microbial exposure, i.e. by comparing peripheral blood B-cells from people living in Indonesia and Ghana to those from healthy Dutch residents. We observed differences in the balance between the naïve and memory compartments, with more atypical and DN MB-cells and less naïve B-cells in individuals from higher pathogen exposure areas compared to residents from an area with less pathogen exposure.

## Materials and methods

### PBMC

Indonesian and Ghanaian samples used in this study come from biorepositories at Leiden University Medical Center (LUMC), the Netherlands, and originated from previous studies on filariasis and schistosomiasis in Indonesia and Ghana, respectively [47, 48]. Indonesian PBMC were collected between 1989 and 1992 on Sumatra island, Indonesia, an area where *Brugia malayi* was endemic [47]. Ghanaian PBMC were collected between 2011 and 2012 in the Greater Accra region of Ghana, an area endemic for *Schistosoma haematobium* [48]. sSEA and sBmA antibody titers and infection status were determined as previously described in the original research articles [47, 48]. PBMC from Dutch children were obtained from the Dutch National Institute for Public Health and Environment (RIVM) and were collected from 2007 onwards in the Netherlands. PBMC from Dutch adult residents were obtained from the LUMC and were collected in 2019. All subjects (or their guardians) gave written informed consent. Research was conducted in compliance with ethical regulations. Ethical approval was given by the RIVM ethics review board (STEG/METC, Almere, the Netherlands, ISRCTN65428640), the LUMC ethics review board (METC, Leiden, the Netherlands), the Institutional Review Board of the Noguchi Memorial Institute for Medical Research, Accra, Ghana, and the Indonesian Department of Health and Human services, Jakarta, Indonesia. Samples were selected based on the availability of the sufficient number of frozen PBMC and cryovials, on age (children below 16 years old, adults between 16 and 40 years old), and absence of elephantiasis, malaria infection, or anemia. Patient information can be found in Table 1. PBMCs were isolated on a Ficoll density gradient and cryopreserved in liquid nitrogen until use [47–49]. PBMC from children (RIVM) were stored at −135°C, while all other samples were stored in liquid nitrogen.

**Table 1:** characteristics of study cohorts. Samples with less than 1000 cells after processing were excluded from the analysis and are not displayed here

Characteristics - children cohort	Europe	Ghana	
*N*	6	18	
Sex, female (*n*, %)	0 (0%)	7 (38.8%)	
Age, years (median, min, max)	9 (9–9)	11.5 (8–15)	
Infected (*n*)*	0	5	
sSEA IgG4, µg/ml (median, min, max)	NA	1028.1 (0.05–1729.5)	

Characteristics - adult cohort	Europe	Indonesia	Ghana
*N*	11	10	12
Sex, female (*n*, %)	4 (36%)	1 (10%)	4 (33%)
Age, years (median, min, max)	26.0 (23 - 29)	23.5 (16–35)	17.5 (16–38)
Infected (*n*) *	0	3	2
sBmA IgG4, µg/ml (median, min, max)	NA	1338.3 (782.2–6978.5)	NA
sSEA IgG4, µg/ml (median, min, max)	NA	NA	1129.8 (534.3–1769.5)

*Infection status of individuals infected with *S. haematobium* in Ghana and microfilaria in Indonesia.

### Cell thawing and B-cell isolation

Cryopreserved PBMCs (5 or 10–10 × 10^6^) were thawed by the addition of a thawing medium (50% HI-FCS in supplemented RPMI-1640 at 37°C). Untouched B-cells were isolated using a negative Pan B-cell isolation kit (#130-101-638, Miltenyi Biotec, Bergisch Gladbach, Germany), following manufacturer’s protocol. The flow-through contained the total B-cell fraction. Cells were resuspended in culture medium (RPMI-1640 plus 10% HI-FCS, 1 mM pyruvate, 2 mM L-glutamine, 100 U/ml penicillin, and 100 μg/ml streptomycin) at 4 × 10^6^ cells/mL and rested overnight at 37°C with 5% CO_2_.

### Cell stimulation

Subsequently, cells were stimulated with 100 ng/ml Phorbol 12-myristate 13-acetate (PMA, Sigma, Saint Louis, USA) and 1 μg/ml Ionomycin (Sigma) in culture medium for 6 h at 37°C. 10 μg/ml Brefeldin A (Sigma) was added during the last 4 h.

### Mass cytometry staining

#### Antibody conjugation

Metal-conjugated antibodies were either purchased or conjugated using 100 μg of purified antibody combined with the Maxpar X8 Antibody Labelling Kit (Standard Bio Tools, San Francisco, USA) according to the manufacturer’s protocol (version 10). Conjugated antibodies (Supplementary Table S1) were stored in Antibody Stabilizer (CANDOR Bioscience, Wangen, Germany) at 4°C.

#### Barcoding of samples

Following stimulation and collection, cells were washed in MaxPar Cell Staining Buffer (Standard Bio Tools). Samples were barcoded randomly in batches of five with a combination of four different metal-conjugated B2M antibodies. Samples from children and adults were processed separately. A reference sample was added to each batch. After 30 min incubation at room temperature (RT) with the appropriate barcoding mix, samples were washed in staining buffer. Samples were pooled by six, with five samples and one reference sample (unstimulated PBMCs).

#### Live/dead staining and surface staining

Pooled cells were incubated with 1 μM Cell-ID Intercalator-103Rh (Standard Bio Tools) in staining buffer for 15 min at RT to identify dead cells. After washing, cells were incubated with a Human Fc receptor blocker (BioLegend, San Diego, California, USA) in staining buffer for 10 min at RT. Subsequently, the surface antibody cocktail was added, incubated for 45 min at RT, and washed twice in staining buffer.

#### Intracellular staining

Following surface staining, cells were fixed and permeabilized using MaxPar Fix I Buffer and MaxPar Perm-S buffer (Standard Bio Tools) and washed twice in MaxPar Perm-S buffer. Cells were incubated with the intracellular antibody cocktail for 30 min at RT. Cells were washed twice with staining buffer, followed by staining with 125 nM Cell-ID Intercalator-Ir (Standard Bio Tools) in MaxPar Fix and Perm buffer (Standard Bio Tools) overnight at 4°C. Thereafter, cells were washed twice with staining buffer and once in MilliQ water. Cells were stored at 4°C until measurement (within 48 h).

#### Compensation beads

A total of 30 μl of compensation beads (BD biosciences, Franklin Lakes, New Jersey, USA) were stained independently with 2 μl of each metal-labeled antibody for 15 min at RT. After 3 washes in staining buffer, beads were pooled together and fixed in Fix and Perm buffer for 1 h at RT. Then, beads were washed twice in staining buffer and resuspended in 500 μl MilliQ water before measurement.

#### Mass cytometry data acquisition

Before measuring, cells were passed over a cell strainer and diluted to 10 × 10^6^ cells/ml with 10% EQ Four Element Calibration Beads (Standard Bio Tools) in Milli-Q water. Samples were measured on a Helios mass cytometer (Standard Bio Tools) using dual-count mode and noise reduction. Next to channels used to detect antibodies, channels for intercalators (103Rh, 191Ir, and 193Ir), calibration beads (140Ce, 151Eu, 153Eu, 165Ho, and 175Lu), and background/contamination (133Cs, 138Ba, and 206Pb) were acquired. After data acquisition, the mass bead signal was used to normalize the short-term signal fluctuations with the reference EQ passport P13H2302.

### Data processing

FCS files were pre-processed using an in-house developed pipeline in R (R x86_64 version 4.1.3, R Foundation for Statistical Computing) and RStudio (Rstudio Inc.). FCS files were normalized using CATALYST package (v1.18.1) [50], followed by debris and doublets removal with CyTOFClean package (v1.0.3) and semi-manual gating on DNA, live/death, and CD45^+^ cells using the openCyto package (v2.6.0) [51, 52].

Subsequently, samples were debarcoded and signal spillover was compensated by CATALYST package Thereafter batch effects were removed using the reference PBMCs with Spectre package (v1.0.0) [53], files were concatenated using premessa package (v0.3.2), and exported as FCS files [54]. After processing samples with less than 10^3^ acquired single cells were removed from the analysis (six samples), resulting in 57 samples that were further analyzed (range 1.228–776.718 single cells per sample).

**Figure 1:**
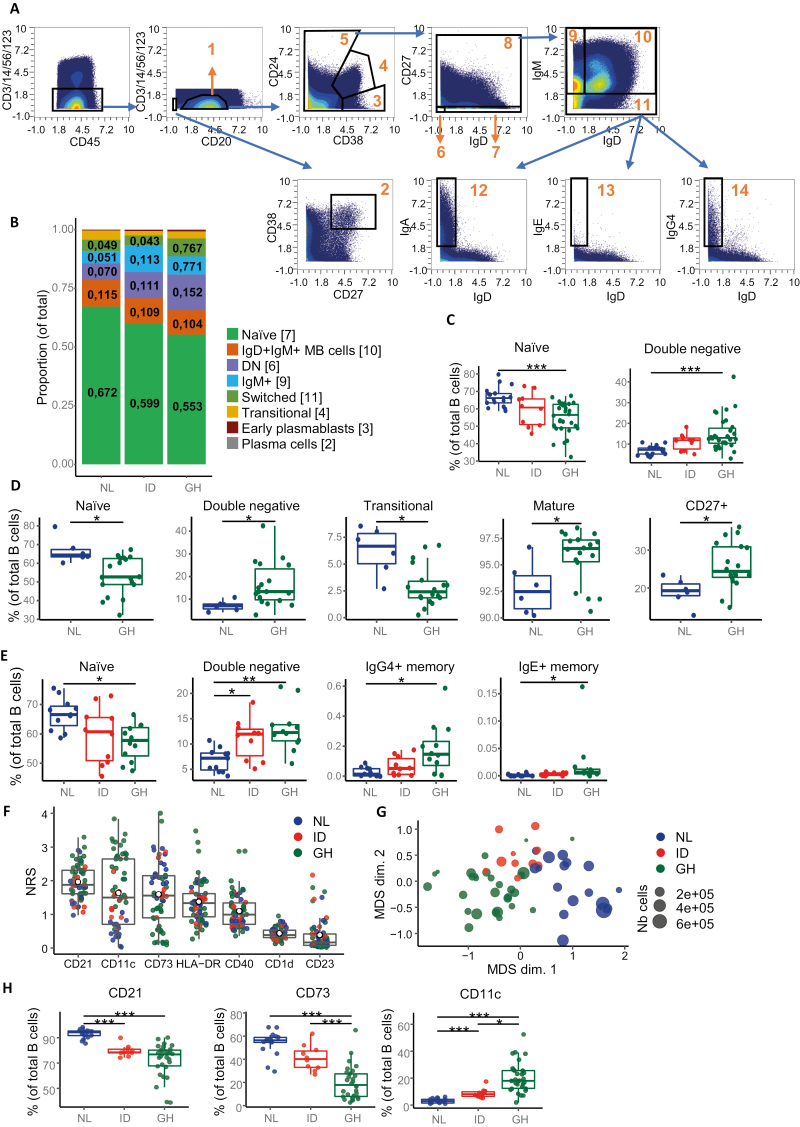
distinct B cell profiles among Dutch, Indonesian, and Ghanaian populations. **(A)** Gating strategy shown in all concatenated files used to manually gate the main B cell populations. Live CD45^+^ CD3^−^ CD14^−^ CD56^−^ CD123^−^ were divided into CD20^+^ B cells (population 1) and CD20^−^ CD27^+^ CD38^+^ plasma cells (population 2). Next, CD20^+^ B cells were separated into CD38^+^ CD24^−^ plasmablasts (population 3), CD38^+^ CD24^+^ transitional B cells (population 4), and CD38^−^ mature B cells (population 5). Mature B cells were further divided into IgD^−^ CD27^−^ double negative (DN) memory B (MB) cells (population 6), IgD^+^ CD27^−^ naïve B cells (population 7), and CD27^+^ MB cells (population 8). Then, CD27^+^ MB cells were separated into IgD^−^ IgM^+^ IgM MB cells (population 9), IgD^+^ IgM^+^ MB cells (population 10), and IgM^−^ switched MB cells (population 11). Finally, MB cells were split according to their immunoglobulin expression into IgA^+^-switched MB cells (population 12), IgE^+^-switched MB cells (population 13), and IgG4^+^-switched MB cells (population 14). **(B)** Stacked bar graph showing the proportion of the main B-cell sub-populations (as determined in 1A) relative to total B cells among Dutch (NL), Indonesian (ID), and Ghanaian (GH) subjects. Brackets indicate the population number from (A). Numbers displayed on the graph indicate the mean proportion. **(C)** Boxplots of the frequencies of naïve and double negative B cells relative to total B cells between countries. Only populations with statistically significant differences are displayed here, see Supplementary Fig. S1A for all B-cell populations. **(D)** Boxplots of the frequencies of various B-cell populations relative to total B cells between Dutch and Ghanaian children. Only populations with statistically significant differences are displayed here, see Supplementary Fig. S1B for all B-cell populations. **(E)** Boxplots of the frequencies of various B-cell populations relative to total B cells among Dutch, Indonesian, and Ghanaian adults. Only populations with statistically significant differences are displayed here, see Supplementary Fig. S1C for all B-cell populations. **(F)** Non-redundancy scores (NRS) listing the variation between each sample for each non-lineage marker in total B cells. Markers used to gate B-cell populations in (A) were excluded from the analysis. Markers on the *x*-axis are sorted according to the decreasing mean NRS. **(G)** Multidimensional scaling (MDS) plot comparing each sample using NR scores. The distance between each point correlates to the dissimilarity between these samples. **(H)** Frequency of CD21^+^, CD73^+^, and CD11c^+^ B cells relative to total B cells. Statistics for (C), (D), (E), and (H) were calculated using a binomial generalized linear mixed model, *P < 0.05, **P < 0.01, ***P < 0.001. Total *n* = 57, Netherlands *n* = 17, Ghana *n* = 30, Indonesia *n* = 10. Total children *n* = 24, Netherlands children *n* = 6, Ghana children *n* = 18. Total adults *n* = 33, Netherlands adults *n* = 11, Indonesian adults *n* = 10, Ghana adults *n* = 12. The analysis involves all pooled samples, including adults and children, unless stated otherwise

Data was transformed using a hyperbolic arcsinh with a cofactor of 5 [55] in OMIQ (Omiq, Inc, Boston, Massachusetts, USA). Next, cells were manually gated to define the major B-cell subsets (Fig. 1A) [56]. An optimized t-distributed Stochastic Neighbor Embedding (optSNE) was performed using OMIQ, where gated subpopulations were downloaded as FCS files and integrated into our R pipeline. Clustering was performed using PARC clustering as implemented in the PARC package [57] and a minimum cluster size set to 500. Of note, this does not apply to cells from individuals in a cluster, but to the merged cells from all individuals. *K* = 50 was used to generate a KNN graph from the low-dimensional embeddings and 15 iterations of the Leiden algorithm.

Clusters produced via manual gating and PARC clustering were analyzed and visualized in R, using the CATALYST and ggplot2 (v3.3.5) packages, producing NRS scores across samples, boxplots, radar plots, and heat maps [50, 58]. Histograms were plotted using OMIQ.

### Statistical analysis

Statistical analyses were performed in R using the lme4 package (v1.1-29) [59]. Statistical differences in abundances of gated subpopulation and clusters between different countries were compared with a binomial generalized linear mixed model with a correction for the total number of analyzed cells per sample used as weights and controlled using the bobyqa optimizer. Individuals were included as a random effect to prevent underdisperion, while country was added as a fixed effect. Integer scalar was changed to 0 when the model failed to converge at the default setting of 1. Post hoc comparisons of all groups were conducted using the emmeans package (v1.7.4-1) with FDR correction for multiple corrections [60]. FDR-corrected *P* values < 0.05 were considered statistically significant. Data is reported as median (IQR) or mean ± SD.

## Results

### Distribution of B-cell subsets

We investigated peripheral blood B-cell frequencies in people living in Ghana, Indonesia, and the Netherlands (Table 1) following 6 h-stimulation with PMA/ionomycin/Brefeldin using a 36-marker mass cytometry panel (Supplementary Table S1).

The main B-cell populations were manually gated (Omiq) on 8.2 million live CD45^+^CD3^−^CD14^−^CD56^−^CD123^−^ cells from all donors using CD20, CD38, CD24, CD27, IgD, IgM, IgA, IgE, and IgG4 according to the gating strategy depicted in Fig. 1A.

First, leftover CD20^−^CD27^hi^CD38^hi^ plasma cells (population #2) were removed from the CD20^+^ B-cells (#1). Next, CD20^+^ B cells were separated in CD19^−^CD20^low^CD38^+^ early plasmablasts (#3), CD38^+^CD24^+^ transitional B-cells (#4), and CD38^−^ mature B-cells (#5). Mature B-cells were further divided into IgD^−^CD27^−^ double negative (DN) memory B-(MB) cells (#6), IgD^+^CD27^−^naïve B-cells (#7), and CD27^+^ MB cells (#8). Next, CD27^+^ MB cells were separated into IgD^−^IgM^+^ IgM MB cells (#9), IgD^+^IgM^+^ MB cells (#10), and IgM^−^ switched MB cells (#11). Finally, MB cells were split according to their immunoglobulin expression into IgA^+^-switched MB cells (#12), IgE^+^-switched MB cells (#13), and IgG4^+^-switched MB cells (#14). IgG^+^ switched B-cells were characterized indirectly as IgM^−^ IgA^−^ IgE^−^ IgG4^−^ switched memory B-cells. To summarize, canonical B-cell subsets were identified: naïve (#7), transitional (#4), non-switched memory (#6, 9, and 10), and switched MB cells (#11, 12, 13, and 14). Naïve B-cells formed the largest group, followed by either IgD^+^IgM^+^ MB cells, DN B-cells, or IgM MB-cells, switched MB-cells, transitional B-cells, plasmablasts, and plasma cells (Fig. 1B). IgA^+^, IgE^+^, and IgG4^+^ MB-cells had the lowest frequency (less than 1%) and were included in the switched MB-cells. Unattributed CD20^+^, mature and CD27^+^ MB-cells were not included (<1% of each population) in the graph. Overall, we observed 1.2-fold fewer naïve B-cells in Ghanian people compared to Dutch people (NL: median = 66.18% (IQR = 5.33%), GH: 56.42% (13.52%); Fig. 1C), while the frequency of DN MB-cells was 1.8-fold higher in Ghanaian compared to Dutch people (NL: 7.2% (2.89%), GH: 13.03% (7.24%); Fig. 1C). Of note, B-cell subset frequencies from Indonesian subjects were mostly in between those from Ghana and the Netherlands and were not statistically different to either one of the countries. The frequencies of other B-cell subsets were not different between the groups (Supplementary Fig. S1A).

When examining children and adults separately, both naïve B-cells and DN MB-cells showed similar differences as for the combined groups (Fig. 1C and D). Additionally, transitional B-cells were found to be lower in Ghanaian children compared to Dutch children, while mature (#5) and CD27^+^ (#8) B-cells were more abundant (Fig. 1D). These findings may reflect a faster development of the B-cell memory compartment in children living in areas with high microbial exposure compared to Dutch children. Interestingly, these differences were not present anymore in adulthood. However, in adults the frequency of IgG4^+^ and IgE^+^ switched MB-cells was higher than in Ghana (Fig. 1E), which aligns with chronic and/or repeated exposure to pathogens, particularly to parasites [61, 62].

Next, we performed a non-redundancy comparison (NRS) to determine which markers (excluding markers used for gating in Fig. 1A) were most dominantly driving differences between countries (Fig. 1F). We observed a ranking in which CD21, CD11c, and CD73 formed the top 3, followed by HLA-DR, CD40, CD1d, and CD23 (Fig. 1G). This was confirmed by a lower percentage and expression level of CD21^+^ and CD73^+^ in B-cells from Ghanaian and Indonesian subjects compared to Dutch subjects, independent of age cohort (Fig. 1H; Supplementary Fig. S1D and E). In contrast, CD11c^+^ in B-cells was 2.7-fold higher in Indonesian and 6-fold in Ghanaian compared to Dutch subjects (NL: 2.97% (2.77%), ID: 8.17% (3.36%), GH: 17.98% (13.16%); Fig. 1H; Supplementary Fig. S1D). Therefore, we conclude that the expression of CD21, CD73, and CD11c defined cell populations that varied the most between countries. CD73, despite significant change does not provide clues for unique populations or features within our data.

Overall, individuals from Indonesia and Ghana had fewer naïve B-cells and more DN MB-cells and CD11c^+^ B-cells, combined with lower CD21 and CD73, compared to Dutch residents.

### Expansion of memory-like CD11c+ B-cells in people from Ghana and Indonesia

As CD11c was ranked in the top 3 of the NRS analysis (Fig. 1F) and a dominant factor explaining the differences between the three countries, we investigated the distribution of CD11c among the different B-cell subsets. Although CD11c can be expressed by activated naïve B-cells [15, 63], it is mostly expressed by atypical memory B-cells, which expand in the context of chronic infections, as well as in several autoimmune diseases [19, 34, 64].

**Figure 2:**
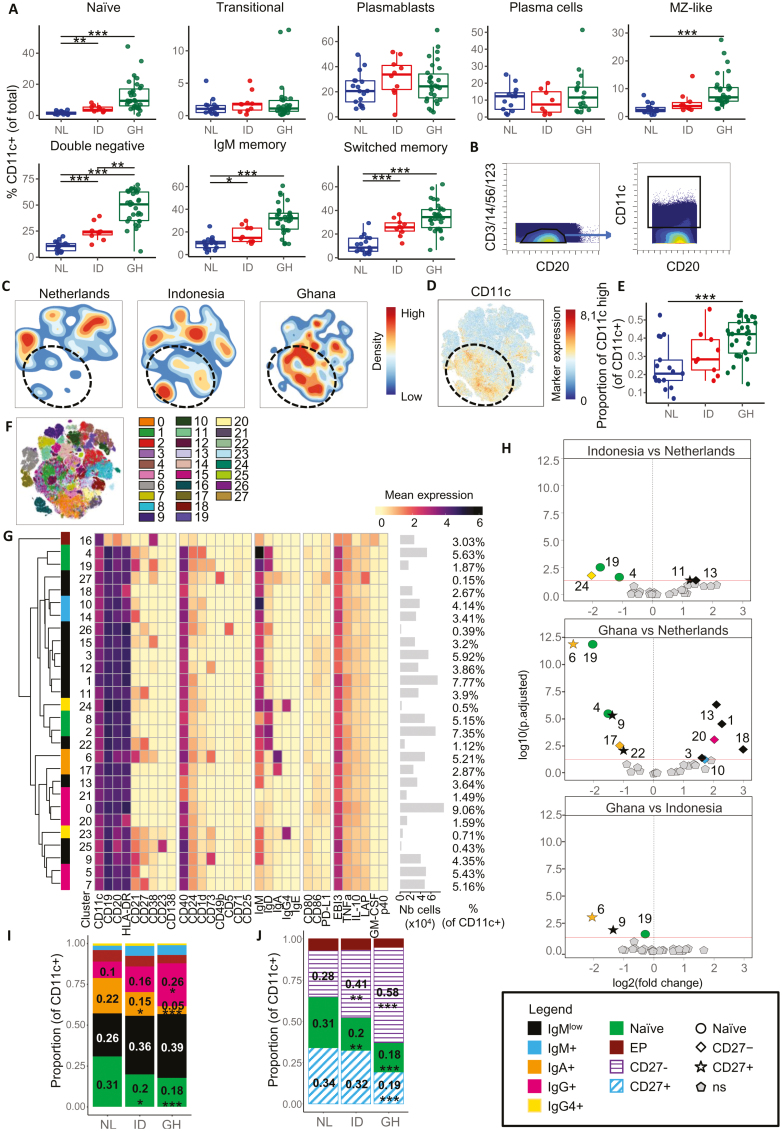
expansion of memory-like CD11c+ B cells in people from Ghana and Indonesia. **(A)** Boxplots of the percentage of CD11c^+^ cells in different B-cell lineages, relative to total B cells, and comparison among the Netherlands (NL), Indonesia (ID), and Ghana (GH). **(B)** Gating of CD11c^+^ B cells out of live CD45^+^ CD3^−^ CD14^−^ CD56^−^ CD123^−^ CD20^+^ cells. **(C)** Density plots on opt-SNE of B cells from Dutch, Indonesian, or Ghanaian subjects. Density is indicated by color. Black dashed ovals highlight CD11c^high^ populations, as shown in (D). **(D)** Opt-SNE of CD11c expression of CD11c^+^ B cells. Black dashed oval highlight the CD11c^high^ population. **(E)** Boxplot comparing the proportion of CD11c^high^ B cells out of CD11c^+^ B cells between the three countries. **(F)** opt-SNE color coded by cluster partition obtained by PARC clustering using all markers except cytokines on all samples. **(G)** Heatmap summary of median expression values of cell markers for all samples colored for arcsin5-transformed expression of the indicated markers. Clusters were color coded according to several main B-cell populations: naïve B cells (clusters 2, 4, 8, 19) (green), early plasmablasts (cluster 16) (EP, dark red), IgMlow B cells [1, 3, 9, 11–13, 15, 18, 22, 25–27] (black), IgM+ (blue) memory B (MB) cells (cluster 10, 14), IgA+ (cluster 6, 17) (orange), IgG + (IgM^−^ IgA^−^ IgE^−^ IgG4^−^); clusters 0, 5, 7, 20, 21 (pink) or IgG4+ (cluster 23, 24) (yellow) switched MB cells. Hierarchical clustering shows the similarity between clusters. Number of cells per cluster and frequency of clusters relative to total CD11c^+^ B cells are indicated on the right side. **(H)** Volcano plots comparing the abundance of CD11c^+^ B cell clusters between Indonesian and Netherlands, Ghana and Netherlands, and Ghana and Indonesia. The log2(fold change) indicates the mean abundance for each cluster and is plotted against log10(adjusted *P* value). In addition to the color codes used in (G), clusters were separated between naïve (circle), CD27− (diamond) and CD27+ (star) cells. Non significatively different clusters are in light gray pentagons. For each volcano plot comparing country 1 with country 2, clusters on the right side of the plot are higher in country 1 compared to country 2, and clusters on the left side are lower in country 1 compared to country 2. **(I)** Stacked bar graph showing the balance between the main different B cell lineages in CD11c^+^ B cells in all three countries. Clusters are color coded as detailed in (G). Mean proportions are indicated on each compartment, as well as statistics comparing ID and GH to NL. Statistics were calculated using medians, and not means, and are only displayed here for easier understanding. Median proportions are visualized in box plots in Supplementary Fig. S2H. **(J)** Stacked bar graph showing the balance between naïve B cells and early plasmablasts in CD11c^+^ B cells in all three countries, as explained in (I). Additionally, CD27− B cells (clusters 0, 1, 3, 5, 10, 12, 13, 14, 15, 17, 18, 20, 21, 24, 27) are indicated with purple stripes and CD27+ B cells (clusters 6, 7, 9, 11, 22, 23, 25, 26) are represented by blue strikes. Median proportions are visualized in box plots in Supplementary Fig. S2I. Statistics for (A), (E), (H), (I), and (J) were calculated using a binomial generalized linear mixed model, *P < 0.05, **P < 0.01, ***P < 0.001. Total *n* = 57, Netherlands *n* = 17, Ghana *n* = 30, Indonesia *n* = 10. Total children *n* = 24, Netherlands children *n* = 6, Ghana children *n* = 18. Total adults *n* = 33, Netherlands adults *n* = 11, Indonesian adults *n* = 10, Ghana adults *n* = 12. The analysis involves all pooled samples, including adults and children, unless stated otherwise

Here, CD11c^+^ B-cells were found in both naïve and MB-cell compartments. Naïve, IgD^+^IgM^+^ MB cells [65, 66], DN, IgM memory, and switched memory B-cells from Ghanaian and/or Indonesia people had more CD11c^+^ expression compared to those from Dutch people, but not plasmablasts, plasma cells, or transitional B-cells (Fig. 2A).

Subsequently, CD11c^+^ B-cells were gated from total CD20^+^ B-cells (Fig. 2B) [55] and density plots (Fig. 2C) showed almost opposite clouds in Dutch and Ghanaian subjects, while Indonesian subjects showed clouds with overlapping features of both the Netherlands and Ghana. Interestingly, for Ghana the cloud corresponded mostly to cells expressing high levels of CD11c (CD11c^high^), as visualized on opt-SNE (Fig. 2D) and in boxplots (Fig. 2E).

PARC clustering on all 8.3 × 10^5^ CD11c^+^ B-cells identified 28 different clusters (Fig. 2F), for which the characteristics are represented in a heatmap (Fig. 2G). Of note, cytokines, activation and exhaustion markers (IL-10, TNFα, GM-CSF, LAP (TGFβ), EBI3, IL-12p40, CD80, CD86, PD-L1) were excluded from the definition of the clustering. Each cluster was assigned to one of seven major B-cell lineage based on key lineage markers and visualized in the heatmap: naïve B-cells (CD19^+^CD20^+^IgD^+^IgM^med/+^; clusters 2, 4, 8, 19), early plasmablasts (CD19^−^CD20^low^CD38^+^; cluster 16), IgM^+^ MB-cells (unswitched or pre-switched) (CD19^+^CD20^+^IgD^−/low^IgM^+^; clusters 10, 14) and switched MB-cells (IgA^+^; cluster 6, 17; IgG4^+^; cluster 23, 24; or IgG^+^(IgM^−^ IgA^−^ IgE^−^ IgG4^−^); cluster 0, 5,7, 20, 21), and undefined IgM^low^ B-cells (clusters 1, 3, 9, 11, 12, 13, 15, 18, 22, 25, 26, 27) [10] (Fig. 2G). Interestingly, CD11c^high^ cells were all found in the CD27^−^ MB-cells (Supplementary Fig. S2B), and CD86 expression was higher in clusters with the highest CD11c expression (clusters 0, 1, 3) (Fig. 2G).

When applying generalized linear mixed modeling, 14 out of 28 clusters were significantly different between the three countries and were found both in memory and naïve CD11c^+^ B-cell subsets. Differences between the groups were plotted in a volcano plot (Fig. 2H) and box plots (Supplementary Fig. S2C).

The percentage of two clusters of naïve CD11c^+^ B cells (clusters 4 and 19) was significatively higher in Dutch people compared to Ghanaian and Indonesian people. These two clusters were uniquely characterized by the expression of CD1d. In general, we observed a dichotomous distribution for CD11c^+^ expression with a higher representation in CD1d^+^ naïve (clusters 4, 19), CD27^+^ CD11c^+^ B-cells (clusters 6, 9, 11, 22), IgA^+^ (cluster 6, 17), and IgG4^+^ MB-cells (cluster 24) from Dutch people, while CD11c^+^ clusters within IgM^+^ B-cells (cluster 10) and IgM^low^CD27^−^CD11c^+^ B-cells (clusters 1, 3, 13, 18) were more abundant in Ghana and/or Indonesia. These (IgM^+/low^)CD27^−^CD11c^+^ cells were also negative for CD21. This phenotype corresponds to atypical, tissue-based MB-cell phenotype reported in different contexts, i.e. healthy individuals, people with SLE, or with infections like HIV or malaria [19, 64].

Next, stacked bar plots were generated to visualize the CD11c^+^ B-cells among the seven B-cell lineages and to compare their abundance between countries (Fig. 2I–J), showing similar differences as in the cluster analysis (Fig. 2H): less naïve B-cells and IgA switched MB-cells among CD11c^+^ B-cells (ID: 18.69% (2.88%), GH: 16.61% (5.24%)) compared to people from the Netherlands (28.94% (16.05%)), as well as less IgA switched B cells (ID: 13.97% (5.15%), GH: 4.08% (3.61%), NL: 19.87% (8.09%)) (Fig. 2I). Conversely, a higher proportion of CD11c^+^ B cells could be assigned to IgM^+^ MB and IgG switched MB-cell lineages in Indonesia and Ghana, albeit the later not significatively, suggesting heterogeneity in memory subsets between individuals. Overall, (Fig. 2J) the balance between CD27^−^ and CD27^+^ subsets was altered, with higher proportion of CD27^−^ MB-cells within the CD11c^+^ B-cells in Indonesia and Ghana compared to the Netherlands, and inversely a lower proportion of CD27^+^ MB-cells within CD11c^+^ B-cells. Interestingly, these CD27^−^ cells are also IgD^−^, and could hence be labeled as DN MB-cells.

When separating the analysis for B-cells from children and adults, we found 10 significatively different clusters out of 28 (clusters 1, 3, 4, 6, 9, 13, 17, 18, 19, and 22) in children versus 11 (clusters 0, 1, 4, 6, 7, 9, 13, 19, 20, 21, and 24) in adults. Those clusters were overlapping with the ones significant when comparing all ages together (Supplementary Fig. S2D and G). In adults, two additional clusters of CD11c^+^ B-cells within IgG^+^ MB-cells were higher in Ghanaian compared to Dutch adults (clusters 0 and 7), suggesting a more advanced class-switching B-cell profile (Supplementary Fig. S2E and H).

To summarize, the percentage and expression of CD11c on B-cells is higher in CD1d^+^ naïve and in MB-cells in people from Ghana and Indonesia, suggesting the development of atypical CD11c^+^ MB-cells and more class-switched MB-cells in adults specifically. The balance between the various MB-cells subsets is also different, as indicated by the expansion of the IgD^−^CD27^−^ DN MB-cell lineage in individuals from Ghana and Indonesia.

### Expansion of CD11c+ double negative type 2 B-cells in Ghana and Indonesia

IgD^−^CD27^−^ double negative (DN) B-cells are regarded as exhausted MB-cells based on phenotypic and immunoglobulin repertoire analyses, and increased numbers were demonstrated in various autoimmune diseases [14–16], chronic infections [11, 12] as well as in elderly [67]. Since we found an enhanced proportion of DN B-cells in Ghana and Indonesia (Fig. 1C), we next examined the phenotype of DN B-cells to better understand their function in areas with different pathogen exposures.

**Figure 3:**
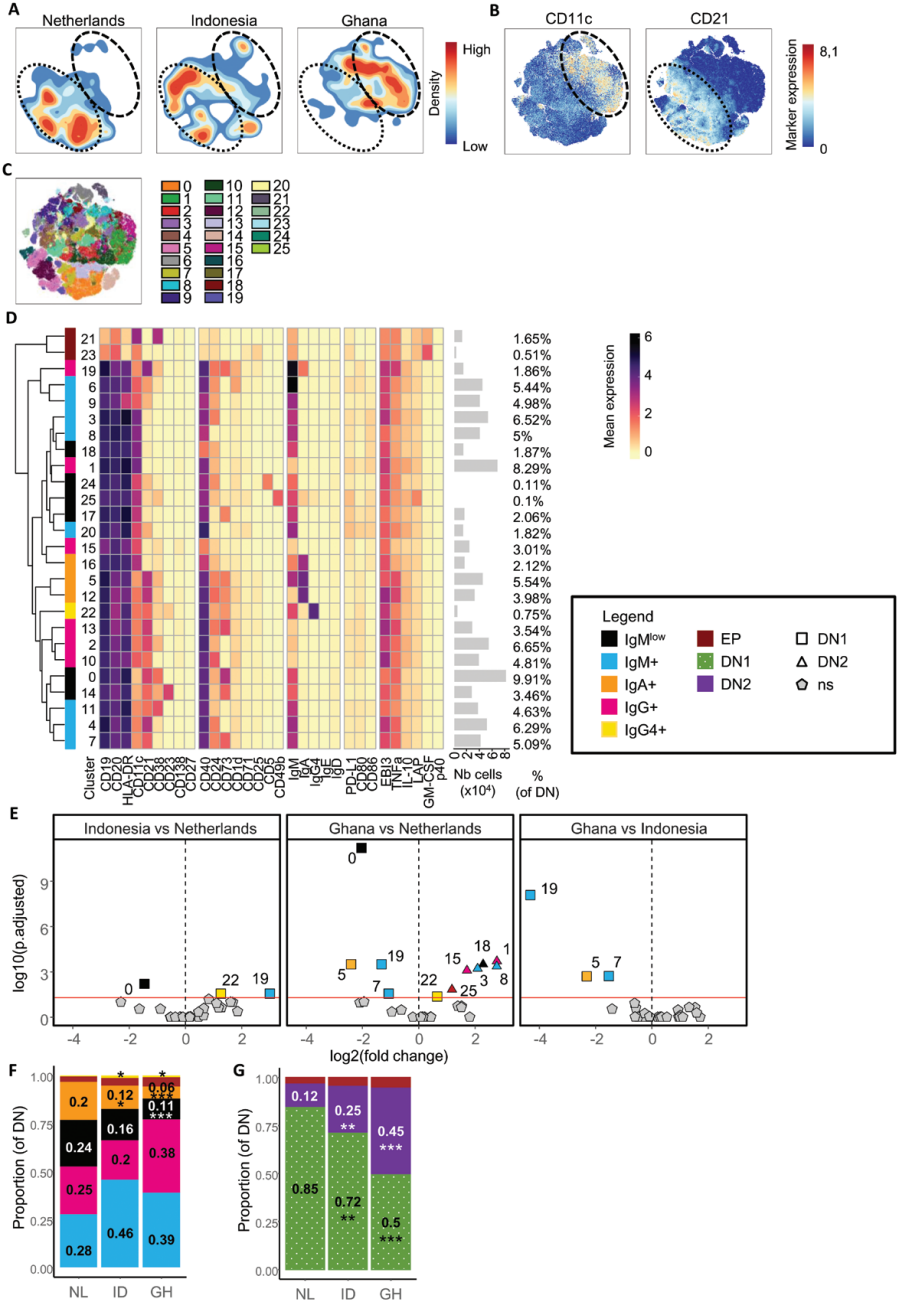
expansion of CD11c+ double negative type 2 B cells in Ghana and Indonesia. **(A)** Density plots of DN B cells of Dutch (NL), Indonesian (ID), or Ghanaian (GH) subjects. Density is indicated by color. Black dashed ovals highlight CD11c^high^ population, while black doted ovals highlight CD21^high^ population as shown in (B). **(B)** Opt-sne of CD11c and CD21 expression in DN B cells. Black dashed ovals highlight CD11c^high^ population, while black doted ovals highlight CD21^high^ population. **(C)** Cluster partition using PARC clustering, which identified 26 different color-coded clusters in all samples. **(D)** Heatmap summary of median expression values of cell markers for all samples colored for arcsin5-transformed expression of the indicated markers. Clusters were color coded according to the several main B cell populations: early plasmablasts (clusters 21, 23) (EP, dark red), IgMlow B cells (clusters 0, 14, 17, 18, 24, 25) (black), IgM+ (blue) memory B (MB) cells (clusters 3, 4, 6, 7, 8, 9, 11, 20), IgA+ (clusters 5, 12, 16) (orange), IgG+ (IgM^−^ IgA^−^ IgE^−^ IgG4^−^); cluster 1, 2, 10, 13, 15, 19 (pink) or IgG4+ (yellow) switched MB cells (cluster 22). Hierarchical clustering shows the relationship between clusters. Number of cells per cluster and frequency of clusters relative to total DN B cells are indicated on the right side. **(E)** Volcano plots comparing the abundance of DN B cell clusters among Indonesian and Netherlands, Ghana and Netherlands, and Ghana and Indonesia, as explained in (H)’s legend. In addition to the color codes used in (D), clusters are separated between DN type 1 (DN, square) and CD11c+ DN type 2 (CD11c+ DN2, triangle) cells. **(F)** Stacked bar graph showing the balance between different B-cell lineages in all three countries. Clusters are color coded as detailed in (D). Mean proportions are indicated on each compartment, as well as statistics comparing Indonesian and Ghanian to Dutch. Statistics were calculated using medians, and not means, and are only displayed here for easier understanding. Median proportions are visualized in box plots in Supplementary Fig. S3H. **(G)** Stacked bar graph showing the balance between different B-cell phenotypes in all three countries, as explained in (F). Additionally, DN1 B cells (clusters 0, 2, 4, 5, 6, 7, 9, 10, 11, 12, 13, 14, 19, 22) are indicated in kaki green with white dots and CD11c+ DN2 B cells (clusters 1, 3, 8, 15, 16, 17, 18, 20, 24, 25) are represented in purple. Median proportions are visualized in box plots in Supplementary Fig. S3I. Statistics for (A), (E), and (H) were calculated using a binomial generalized linear mixed model, *P < 0.05, **P < 0.01, ***P < 0.001. Total *n* = 57, Netherlands *n* = 17, Ghana *n* = 30, Indonesia *n* = 10. Total children *n* = 24, Netherlands children *n* = 6, Ghana children *n* = 18. Total adults *n* = 33, Netherlands adults *n* = 11, Indonesian adults *n* = 10, Ghana adults *n* = 12. The analysis involves all pooled samples, including adults and children, unless stated otherwise

IgD^−^CD27^−^ DN B-cells were gated from CD20^+^CD38^−^ B-cells using OMIQ (Fig. 1A). Two DN B-cells types are recognized: i.e. DN type 1 (DN1), expressing CD21, and DN type 2 (DN2), expressing CD11c, regarded as MB-cell precursors or as extrafollicular antibody secreting cell precursors and atypical/tissue-based MB-cells, respectively [10]. Density plots on DN B-cells (Fig. 3A) revealed distinct fingerprints in the three countries: CD21^+^ DN B-cells (DN1) were most prevalent in Dutch, while CD11c^+^ DN B-cells (DN2) were most abundant in Ghanaian DN B-cells (Fig. 3B).

PARC clustering on 8.5 × 10^5^ gated DN B-cells identified 26 clusters (Fig. 3C). Mean expression of cell markers was summarized in a heatmap (Fig. 3D) and differences between the groups were plotted in volcano plots (Fig. 3E) and boxplots (Supplementary Fig. S3B). In total, 11 out of 26 clusters were significantly different between countries. Indeed and in agreement with the data in Fig. 3A, only CD11c^+^ DN2 B-cell clusters were higher in Ghanaian subjects compared to Dutch ones (clusters 1, 3, 8, 15, 18, and 25), while oppositely, the frequency of CD21^+^ DN1 B-cells was higher in Dutch subjects compared to Ghanaian ones (clusters 0, 5, and 19). For Indonesia, only CD21^+^ DN1 clusters were significatively different from the two other countries (clusters 0, 5, 7, 19, and 22). Overall, the same striking differences in DN1 versus DN2 MB-cells were observed among the major B-cell lineages within DN B-cells (Fig. 3F), including a lower proportion of IgM^low^ MB-cells and IgA^+^ MB-cells in Ghana and/or Indonesia, and a higher proportion of IgG4^+^ MB-cells in Indonesia compared to the Netherlands (Fig. 3E). Interestingly, CD21^+^ DN1 B-cell clusters (0, 5, 7, and 19) in Dutch subjects expressed CD73, which was absent in Ghanaian subjects (also in line with Fig. 1H). DN MB-cells also overlapped with CD21^−^CD27^−^ atypical B-cells (Supplementary Fig. S7).

Separating children from adults, we observed a similar trend as compared to the whole dataset (Supplementary Fig. S3C–G). Ghanaian children and adults had respectively a 1.9- and 1.7-fold higher percentage of DN B-cells within CD20^+^ B-cells compared to Dutch children and adults, respectively (NL children: 7.09% (2.14%) of total B cells, NL adults: 7.2% (3.36%), GH children: 13.34% (13.63%), and GH adults: 12.27% (3.17%),) (Supplementary Fig. S3C). Within the clustered DN B-cells, six clusters were significantly different for adults, while eight clusters were different for children. Among those, one cluster of IgM^+^ MB-cells (cluster 20) was uniquely higher in Ghanaian children, which was not different when evaluating the combined datasets ( Supplementary Fig. S3C). This may suggest that children living in areas with more microbial exposure experience a faster development of their DN MB-cell compartment as compared to Dutch children, and that differences in DN MB-cells profiles may reduce over time.

To summarize, we observed differences in isotype-switched populations in the DN B-cell compartment between the three countries, but the most striking difference is the expansion of CD11c^+^ DN2 B-cells over DN1 cells in Indonesia and Ghanaian people.

### CD11c and CD27 expression drives differences between countries in IL-10 producing Breg cells

B-cells are also involved in the regulation of immune responses, especially through the production of anti-inflammatory cytokines IL-10, TGF-β, and IL-35, as regulatory B-cells (Breg) [29, 30]. Previous studies reported increased IL-10-producing Breg cells in high parasite exposure areas [46, 49], but these cells are less well characterized, as only a few markers were used to define them. Using our panel of 29 B-cell markers, including markers regularly used to define Breg-cell subsets, we performed a deep characterization in people from Ghana, Indonesia, and the Netherlands.

**Figure 4:**
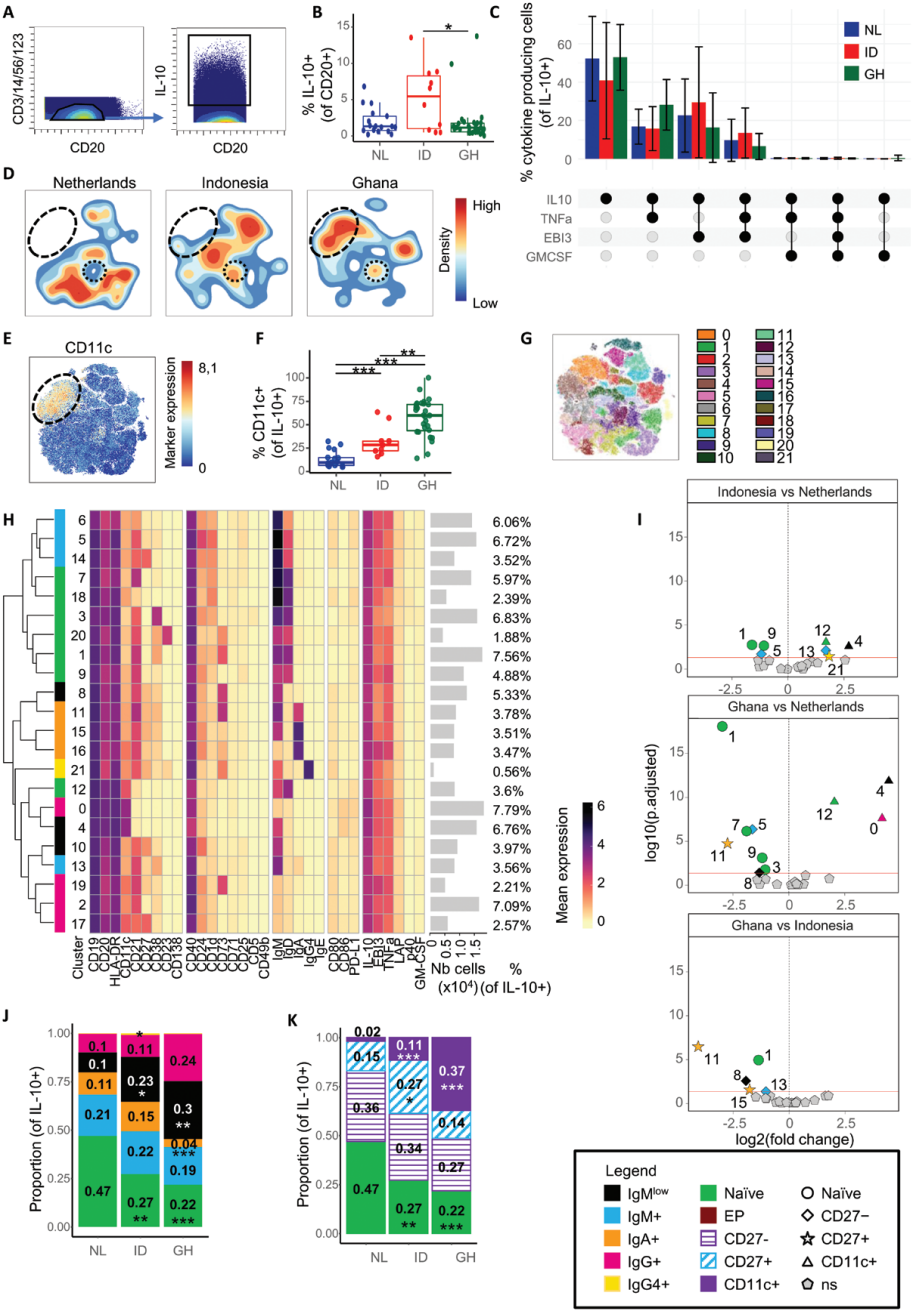
CD11c and CD27 expression drives differences between countries in IL-10 producing Breg cells. **(A)** Gating of IL-10^+^ B cells out of live CD45^+^ CD3^−^ CD14^−^ CD56^−^ CD123^−^ CD20^+^ B cells. **(B)** Boxplots showing the comparison of IL-10^+^ B cell frequencies, relative to CD20^+^ B cells, among Dutch (NL), Indonesian (ID), and Ghanaian (GH) subjects. **(C)** Histograms of the frequencies of cytokine producing cells in IL-10^+^ B cells, and comparison between countries. The cytokine combination produced by each histogram is indicated below the graph. A threshold of at least 100 cytokine producing cells per combination was set. **(D)** Density plots made using opt-SNE of B cells from Dutch, Indonesian, or Ghanaian subjects. Density is indicated by color. Black dashed ovals highlight CD11c^high^ population (see (E)), while black doted circles highlight a naïve subset only present in Indonesia and Ghana. **(E)** Optsne of CD11c expression in IL-10^+^ B cells. Black dashed oval highlight CD11c^high^ population. **(F)** Frequency of CD11c^+^ B cells in IL-10^+^ B cells was compared between countries. **(G)** Cluster partition using PARC clustering, which identified 22 different color-coded clusters in all samples. **(H)** Heatmap summary of median expression values of cell markers for all samples, as detailed in (G). Clusters were color coded according to the several main B-cell populations: naïve B cells (cluster 1, 3, 7, 9, 12, 18, 20) (green) IgMlow B cells (clusters 4, 8, 10) (black), IgM+ (blue) memory B (MB) cells (clusters 5, 6, 13, 14), IgA+ (clusters 11, 15, 16) (orange), IgG+ ((IgM^−^ IgA^−^ IgE^−^ IgG4^−^); clusters 0, 2, 17, 19) (pink) or IgG4+ (yellow) switched MB cells (cluster 21). **(I)** Volcano plots comparing the abundance of IL-10^+^ B cell clusters between Indonesian and Netherlands, Ghana and Netherlands, and Ghana and Indonesia, as explained in (H). **(J)** Stacked bar graph showing the balance between different B-cell lineages in all three countries. See legend for color coding. Mean proportions are indicated on each compartment, as well as statistics comparing ID and GH to NL. Statistics were calculated using medians, and not means, and are only displayed here for easier understanding. Median proportions can be visualized on box plots in Supplementary Fig. S4J. **(K)** Stacked bar graph showing the balance between different B-cell phenotypes in all three countries, as explained in (J). Additionally, CD27− B cells (clusters 5, 5, 6, 8, 13, 16, 19) are indicated with purple stripes, CD27+ B cells (clusters 10, 11, 14, 15, 17, 21) by blue strikes and CD11c^+^ B cells (clusters 0, 4) are represented in purple. Median proportions can be visualized on box plots in Supplementary Fig. S4K. Statistics for (B), (C), (F), (I), (J), and (K) were calculated using a binomial generalized linear mixed model, *P < 0.05, **P < 0.01, ***P < 0.001. Total *n* = 57, Netherlands *n* = 17, Ghana *n* = 30, Indonesia *n* = 10. The analysis involves all pooled samples, including adults and children, unless stated otherwise.

IL-10^+^ B-cells were gated from total CD20^+^ B-cells using OMIQ (Fig. 4A) and the average percentage of IL-10^+^ B-cells was displayed on a boxplot (Fig. 4B). Interestingly, Indonesian people had 4.1-fold more IL-10^+^ B-cells (percentage of total CD20^+^ B-cells) compared to Dutch people (NL: 1.33% (1.96%), ID: 5.43% (7.27%)), while Ghanaian people had slightly less overall IL-10 producing B-cells (GH: 1.13% (1.21%)) than Dutch people. Furthermore, IL-10^+^ B-cells from Indonesian individuals tended to have lower expression levels (MMI) than Dutch and Ghanaian individuals (Supplementary Fig. S4B), suggesting various levels of contribution to the total IL-10 production by those B-cells. There were no statistical differences in the co-producing cytokines of these IL-10^+^ B-cells (Fig. 4C). Nevertheless, the major cytokines co-produced along with IL-10 varied between countries: while IL-10 single producer cells were the most abundant for all three countries (NL: mean ± SD = 52.17 ± 22.04% of total IL-10^+^ B cells, ID: 40.76 ± 30.28%, GH: 52.87 ± 17.12%), the cytokine most often produced together with IL-10 was TNFα in Ghanaian samples (28.19 ± 13.16%), and EBI3 in Dutch and Indonesian samples (NL: 22.57 ± 19.06%, ID: 29.42 ± 28.9%). TNFα is mostly known for its pro-inflammatory capacities [68], but it can also act anti-inflammatory, as it was described to increase Treg expansion and function [69, 70]. EBI3 is a subunit of IL-27, IL-35, and IL-39, which are anti-inflammatory cytokines able to induce IL-10 secretion and reduce inflammatory responses [71–73]. These results suggest that the impact of IL-10 production by B-cells may depend on the total cytokine environment and that the presence of other cytokines may influence the net anti-inflammatory response of these B-cells.

After performing an opt-SNE on the IL-10^+^ cells, density plots of IL-10^+^ B-cells (Fig. 4D) showed distinct fingerprints among the three countries. One of the most obvious differences was the absence of a CD11c^high^ population (Fig. 4E, dashed circle) in Dutch compared to Ghanaian subjects. Boxplots of the percentage of CD11c^+^ cells in IL-10^+^ B-cells confirmed this observation: More than half of IL-10^+^ B-cells in Ghanaian people were also CD11c^+^ (59.83% (27.98%)), 6.3-fold more than in Dutch people (9.51% (8.33%)) and 2.1-fold more than in Indonesian people (28.21% (10.44%)) (Fig. 4F), while Indonesian people had 3 times more CD11c^+^ IL-10^+^ B-cells than Dutch people. This is in agreement with other studies showing increased CD11c^+^ IL-10^+^ B-cells in individuals from tropical rural areas compared to Europeans [34].

We next clustered 2.6 × 10^5^ gated IL-10^+^ B-cells using PARC, and identified 22 different clusters (Fig. 4G). Mean expression of the cell markers was summarized in a heatmap (Fig. 4H). Differences between the groups were plotted in volcano plots (Fig. 4I). From the clusters 13 out of 22 were different between countries. Similar to what was observed for CD11c^+^ (Fig. 2) and DN B-cells (Fig. 3), Dutch people had more naïve IL-10^+^ B-cells than Ghanaian or Indonesian people (clusters 1, 3, 7 and 9), except for cluster 12. Interestingly, this was the only CD11c^+^ naïve cluster. It was characterized by IgM^low^ and absence of CD21 and CD24 and could be a population of activated or anergic B-cells. In accordance with previous results (Fig. 4D and E), CD11c^+^ clusters were higher in Indonesian and Ghanaian subjects (clusters 0, 4, and 12), whether they were IgG^+^ (cluster 0), IgM^low^ (cluster 4) or naïve (cluster 12) B-cells. When evaluating switched MB-cells, IgA^+^ IL-10^+^ B-cells were higher in Dutch individuals (cluster 11), while IgG4^+^ IL-10^+^ B-cells were higher in Indonesian compared to Dutch and Ghanaian people (cluster 21). Overall, only the CD27^+^ switched MB cell clusters (clusters 8 and 11) were different between countries, while CD27^−^ clusters (clusters 10 and 16) were not. Conversely, in (non-switched) IgM^+^ IL-10^+^ MB-cells, only CD27^−^ clusters (clusters 5 and 13), but not CD27^+^ clusters (cluster 14), were different between countries. These results suggest that CD11c^+^ and CD27^+^ in IL-10^+^ B-cells determine most differences between the countries.

Various Breg cell subsets have been described in the literature and can be identified here as well: CD1d^+^ Breg cells (clusters 3, 5, 6, 7, 14, and 18), CD24^hi^CD38^hi^ (cluster 3), CD21^hi^CD23^hi^ (cluster 20), CD21^hi^CD23^−^CD24^hi^IgM^hi^IgD^lo^ (clusters 8, 10, 11, and 15), CD24^hi^CD27^+^ (clusters 8, 10, 11, 15, 16, 17, and 21) and CD11c^+^ (clusters 0, 4, 12, and 17) Breg cells. However, their frequency did not differ between countries.

When analyzing samples from children or adults separately, we observed similar trend similar to those described for the whole dataset (Supplementary Fig. S4G–J), although there were more differences between adults (11 significatively different clusters) than in children (10 significatively different clusters). There were two additional clusters (clusters 6 and 18, IgM^+^ MB-cells and activated naïve B-cells, respectively) higher in Ghanaian and one IgG^+^ CD27^−^ cluster higher in Dutch children (cluster 19), which were not different in the combined datasets. Furthermore, IgA and IgG4 clusters were not different between countries in children, while they were in adults and/or combined datasets. As observed previously with CD11c^+^ and DN B-cells, these results may suggest a faster/ accelerated B-cell development/maturation in children from areas with a higher environmental exposure, but these differences likely lessen upon aging as adults from both areas were more similar in this respect.

The anti-inflammatory immumoglobulin IgG4 was linked to IL-10-producing Breg cells [74]. It was shown that IL-10-producing CD27^−^ Br1 cells (CD73^−^CD25^+^CD71^+^) produced [allergen-specific] IgG4 following *in vitro* stimulation [74]. However, another study suggested that IL-10^+^ B-cells did not develop into IgG4-producing plasma cells and that IgG4 was predominantly produced by non-IL-10-expressing cells [75]. In our samples, there were very few IgG4- and IL-10-co-producing B-cells (Supplementary Fig. S5A). Ghanaian people had a 1.6-fold higher percentage of IgG4^+^ B-cells than Dutch people (NL: 0.2% (0.15%) of CD20^+^ B cells, GH: 0.33% (0.23%)) (Supplementary Fig. S5B), but this increase came from IL-10^−^ B-cells, and not from IL-10^+^ B-cells (Supplementary Fig. S5C). This suggests that IgG4 and IL-10 are not necessarily co-produced by the same B-cell. However, we cannot exclude that (subsets of) IL-10-producing B-cells could develop into IgG4-producing plasma cells.

Overall, we observed statistical differences between various clusters of IL-10-producing B-cells, in which CD11c and CD27 seemed to be most able to discriminate IL-10^+^ B-cell subsets between the three countries, and those subsets may play a distinct role in the immune response towards pathogens.

## Discussion

Our study reveals a high-dimensional overview of different B-cell populations in individuals living in rural tropical areas as compared to subjects living in the Netherlands, allowing us to investigate the role of environmental exposure and living circumstances on B-cell dynamics. Using a 36-marker mass cytometry panel, we observed differences in the balance between the naïve and memory compartments, with higher CD11c^+^ and DN MB-cells in individuals from rural tropical areas, and conversely lower naïve B-cells. Furthermore, characterization of total B-cell populations, CD11c^+^, DN, and Breg cells showed the emergence of specific memory clusters in individuals living in rural tropical areas.

The NRS showed that CD21, CD11c, and CD73 were mainly driving differences between countries. CD21 and CD73 were higher in Dutch people, while CD11 was higher in Ghanaian and Indonesian people. In other studies, decreased CD21 and CD73 was also found with chronic antigen stimulation and chronic infections [76–78]. CD73 is an ectonucleotidase that generates adenosine (ADO) from AMP. Decreased CD73 expression and ADO production were linked to increased B-cell activation and inhibition of T-cell proliferation and cytokine production, potentially restricting the harmful effects of activated T cells [78–80]. The lower frequency of CD21^+^ B-cells, and conversely the higher percentage of CD21^−^ B-cells, is of interest here due to its relationship with CD21^−^CD27^−^ B-cells (atypical B-cells) and with IgD^−^CD27^−^ DN B-cells. There is an overlap in the literature between CD21^−^CD27^−^ B-cells and CD11c^+^ B-cells, referring to them as atypical B-cells or IgD^−^CD27^−^ DN B-cells interchangeably. We agree there is some overlap between the populations, but they can also be gated separately (Supplementary Fig. S7). Here, we observed that CD21^−^CD27^−^ B-cells shared the most cells with other populations, as only ~25% of CD21^−^CD27^−^ did not also belong to CD11c^+^ or DN B-cells. CD11c^+^ was the most diverse and separate population, with ~50% of CD11c^+^ not being part of the other two gates. Interestingly, almost half of CD11c^+^ B-cells were also CD21^−^CD27^−^, which is in accordance with findings in malaria infections showing increased CD11c^+^ B-cells [13] with ~40% of CD11c^+^ B-cells also displaying this atypical B-cell phenotype. One-third of CD11c^+^ were also IgD^−^CD27^−^, qualifying them as DN2 B-cells.

The exact nature of these non-classical MB-cells is still under investigation. The main hypothesis is that these cells are exhausted B-cells, which is supported by their hyporesponsive to BCR or TLR stimulation, their increased expression of inhibitory receptors, and their stunted Ig diversity [19, 64, 81, 82]. However, the fact that already Ghanaian children had significatively more CD11c^+^ B-cells than adults suggests that there could be more than the role of exhausted B-cells. As these non-conventional MB-cells can be stimulated through antigen presented on membranes [83], it has been proposed that they could represent a transitional stage between activated B-cells (indicated by an increased expression of activation markers such as CD86 or CD69 (18)) and short-lived plasma cells, developing through an extrafollicular differentiation pathway and not the GC pathway [15]. The lack of proper selection outside of the GC would suggest an enrichment of autoreactive clones in these cells [84, 85]. This seems counterintuitive, as our study, as well as others [34], shows an increased proportion of CD11c+ and DN2 B-cells in individuals from rural tropical areas and these areas are in fact associated with a lower prevalence of autoimmune diseases. This discrepancy might be due to the context: studies have shown that atypical B-cells in autoimmune diseases produced proinflammatory cytokines and autoreactive antibodies [84, 85], whereas atypical B-cells associated with HIV or malaria infections did not always differentiate into antibody-producing plasma cells. The ones that did differentiate exhibited a markedly reduced cytokine and antibody production capacity [81, 82]. This suggests that atypical B-cells that arise in the context of chronic infections versus autoimmunity may differ functionally, as well as phenotypically. Due to the increased frequencies of IL-10-producing CD11c^+^ B-cells in individuals from rural tropical areas observed here and by others [34], we favor the hypothesis of an immunomodulatory role for atypical CD11c^+^ cells. The enrichment in CD11c^+^ B-cells observed in chronic infections, could be due to more exhausted B-cells and/or to more immunomodulatory B-cells, explaining the difficulty to eliminate the pathogen and the establishment of a chronic infection. Overall, studies have suggested diverse roles for DN B-cells, CD21^−^CD27^−^ atypical B-cells, and CD11c^+^ B-cells, but the exact nature and relationship are not yet fully understood, and more research is needed.

To study Breg cells between different living areas, we measured the expression of immunoregulatory cytokines (IL-10, LAP, and EBI3), as well as CD1d, CD24, CD38, CD40, CD73, CD71, CD25, CD5, CD49b, markers used to characterize Breg subsets in the literature [29–31]. Here, the frequency of IL-10^+^ B-cells was not different between individuals from the Netherlands, Ghana, and Indonesia, although there was a trend towards more IL-10^+^ B-cells in Indonesia. However, we have previously reported more IL-10^+^ B-cells or IL-10^+^CD1d^+^ B-cells in *S. haematobium*-infected young adults compared to non-infected controls from endemic or non-endemic areas [28, 49], but these cells were restimulated *in vitro* with LPS and PMA, ionomycin and Brefeldin A (PIB), or by helminth antigens and anti-IgM/IgG, while here we only restimulated with PIB. Furthermore, we have not evaluated total IL-10 production by ELISA and differences may not have been noted applying intracellular labeling only [86] or may be related to specific subsets only (as suggested here). This may imply that increased IL-10 production by (CD1d^+^) B-cells may only be visualized within antigen-specific recall responses or specific adjuvants that are known for their IL-10 inducing capacity in B-cells (e.g. LPS [87]). In another study, children infected with *S. haematobium* had higher percentages of IL-10^+^ B-cells compared to uninfected children, but this effect was gone after treatment, suggesting that Breg cell development and activity is more transient and subject to either specific helminth infection or living circumstances, explaining the lack of differences as described here [28].

The differences we observed in B-cells between populations from Europe, Africa, and South-East Asia may not only influence protective immune responses towards pathogens but also towards vaccines. Indeed, immune response after vaccination as well as vaccine protective efficacy can differ among countries, populations, or ethnicities, as shown by studies on HIV [88], COVID-19 [89], BCG [33, 90], and yellow fever vaccines [91, 92]. This indicates that it is essential to better understand the effect of living environments on the immune system and the development of protective responses in order to improve vaccine development and reduce disparity in vaccine protective efficacy between populations [93].

We observed that children from rural tropical areas had a more “advanced” B-cell development profile compared to Dutch children and that differences were less pronounced in adults. There is a stereotypic pattern of immune system development that is shared and followed by all children [94] and which is influenced by age [85]. Early-life exposure mostly influences the developmental pathway of the immune system [95–97]. In the first few years of life, microbial exposure is a strong driver of the stereotypic development of the immune system [94]. Many studies have linked environmental exposure differences, such as pre/postnatal exposure to various infections, with altered immune responses, e.g. towards vaccines, in children and adults [98]. Our results suggest that a putative higher exposure to pathogens due to rural and tropical living circumstances will accelerate the development of the immune system. A similar development will also occur in regions with lower microbial exposure and different living conditions (such as in the Netherlands), only slower and/or later in life, creating a lag time during childhood that may be less pronounced in adulthood. It should be noted, however, that Dutch children were slightly younger compared to Ghanian children and we had a gender bias that may have influenced our results.

Our study’s limitations include a lack of information on dwelling residency and area, a relatively small number of samples with a wide range in collection time (stretching several decades), differences in age and gender between the different countries, the absence of samples from Indonesian children and limited diagnostics on common parasitic infections which may have confounding effects on our results. However, given the endemic status of some of these parasites, we can regard the data obtained here as “real world” data and representative of the everyday life of people living in tropical rural areas. Therefore, the heterogeneity for parameters such as infection status or disease history is both a limitation and a strength, as the greater variability may affect statistical outcomes negatively, but is a good reflection of environmental exposure and living circumstances. Unfortunately, we did not have samples from Dutch residencies living in rural areas. Lastly, a marker for IgG is lacking in our study due to technical issues with the pan-IgG marker used in the CyTOF panel and we had to gate and phenotype IgG^+^ B-cells in an indirect manner, as B-cells negative for IgM, IgA, IgG4, or IgE.

To summarize, we report differences in naïve, CD11c^+^, DN, and IL-10^+^-producing B-cells between individuals living in the Netherlands, Indonesia, and Ghana: i.e. increased frequency of CD11c^+^ and double negative B-cells (possibly representing exhausted B-cells) in Ghanaian and Indonesian people. These differences were most evident during childhood and partly persisted in adulthood. We hypothesize this is due to differences in microbial exposure in living environment. Considering the importance of B-cells for protective immunity, these differences may explain geographical variations in vaccine responses and the frequency of hyperinflammatory disorders (such as allergies or autoimmunity).

## Supplementary Material

uxae074_suppl_Supplementary_Figures

uxae074_suppl_Supplementary_Data

## Data Availability

The data underlying this article will be shared on reasonable request to the corresponding author.

## Abbreviations

AAM: alternative activated macrophages
ADO: ectonucleotidase, generating adenosine
BCRs: B-cell rec
Breg: regulatory B-cells
CD: cluster of differentiation
DN: double negative
EBI3: Epstein-Barr virus-induced gene 3
GC: germinal centereptors
GH: Ghana
ID: Indonesia
GM-CSF: granulocyte-macrophage colony-stimulating factor
IL-10: interleukin 10
IL-35: interleukin 35
LAP: transforming growth factor beta
MB-cells: memory B-cells
NL: Netherlands
NRS: non-redundancy comparison
optSNE: optimized t-distributed Stochastic Neighbor Embedding
PB: plasmablasts
PC: plasma cells
TNFα: tumor necrosis factor alpha
Treg: regulatory T-cells
