# Supplementary material for: Multi-dimensional analysis of B cells reveals the expansion of memory and regulatory B-cell clusters in humans living in rural tropical areas
Source: Clin Exp Immunol. 2024 Aug 12;219(1):uxae074. doi: 10.1093/cei/uxae074 (PMC11771192; doi:10.1093/cei/uxae074)
Supplement: uxae074_suppl_Supplementary_Figures [file uxae074_suppl_Supplementary_Figures.pdf]

Supplementary figures

- 1: all B cells, box plots subsets abundance, all data, adults and children
- 2: CD11c+, box plots subsets abundance, all data, adults and children
- 3: DN, box plots subsets abundance, all data, adults and children
- 4: IL10+, box plots subsets abundance, all data, adults and children
- 5: cytokine gating

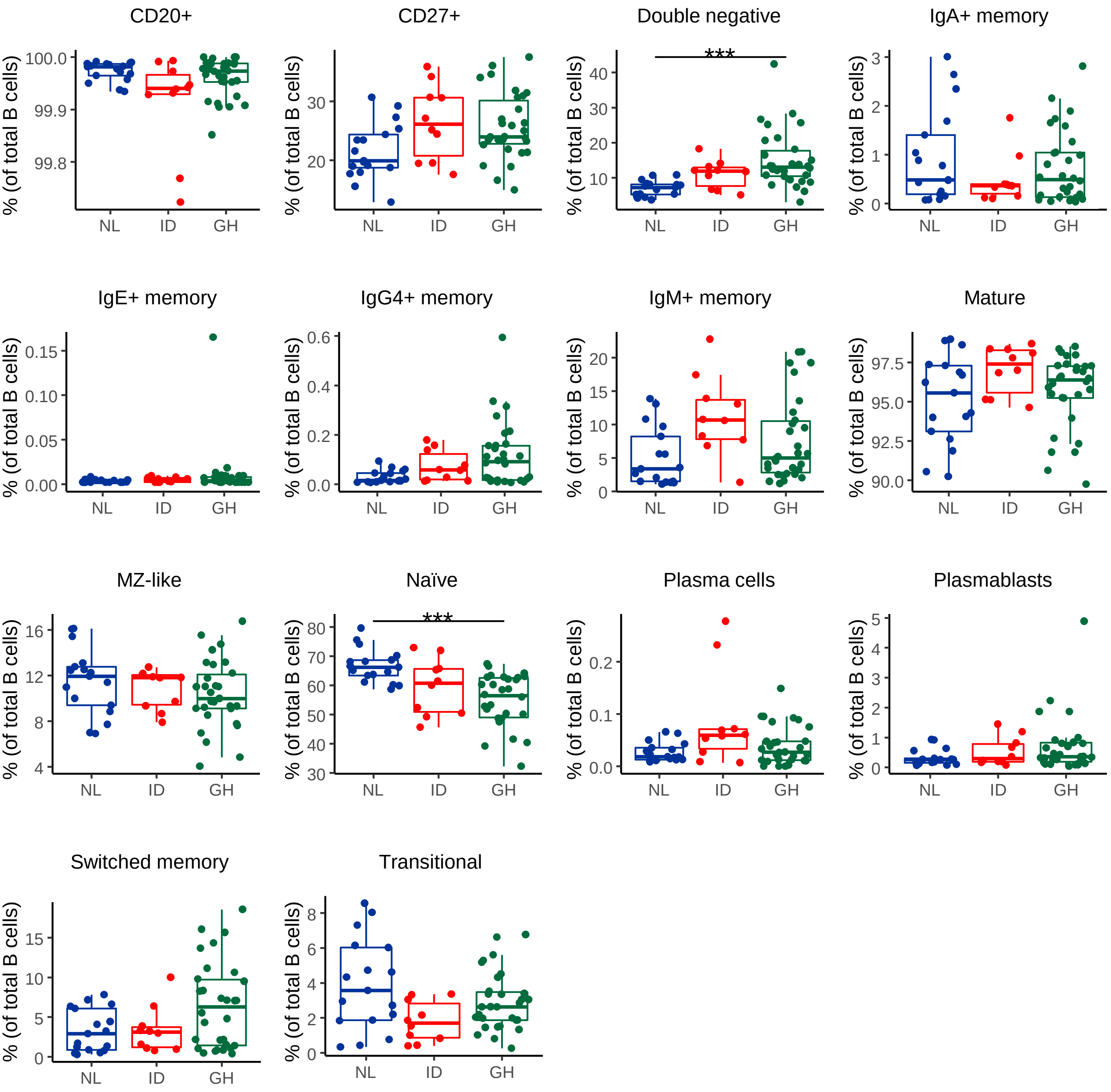

Supplementary Figure 1A:

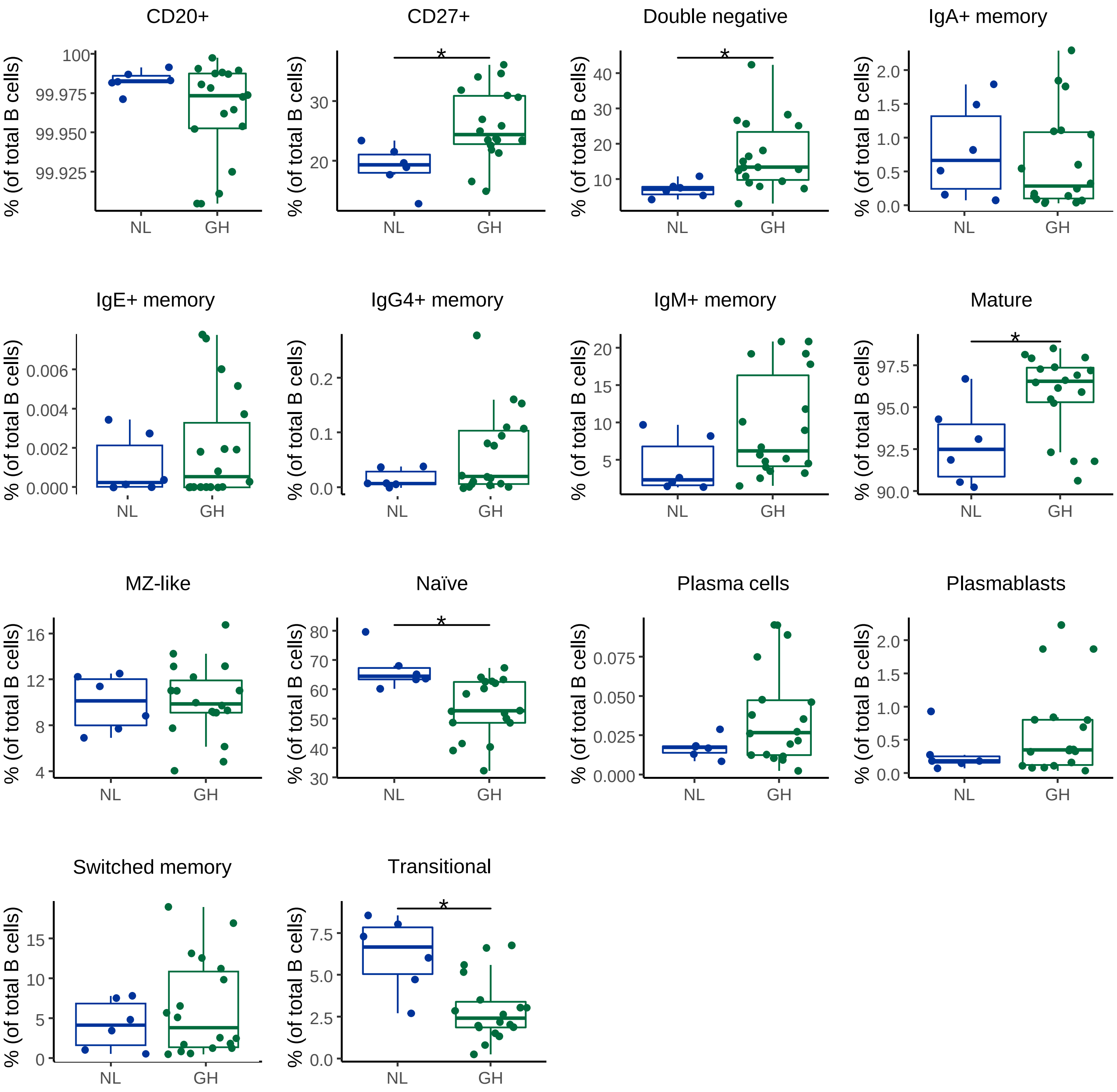

Supplementary Figure 1B:

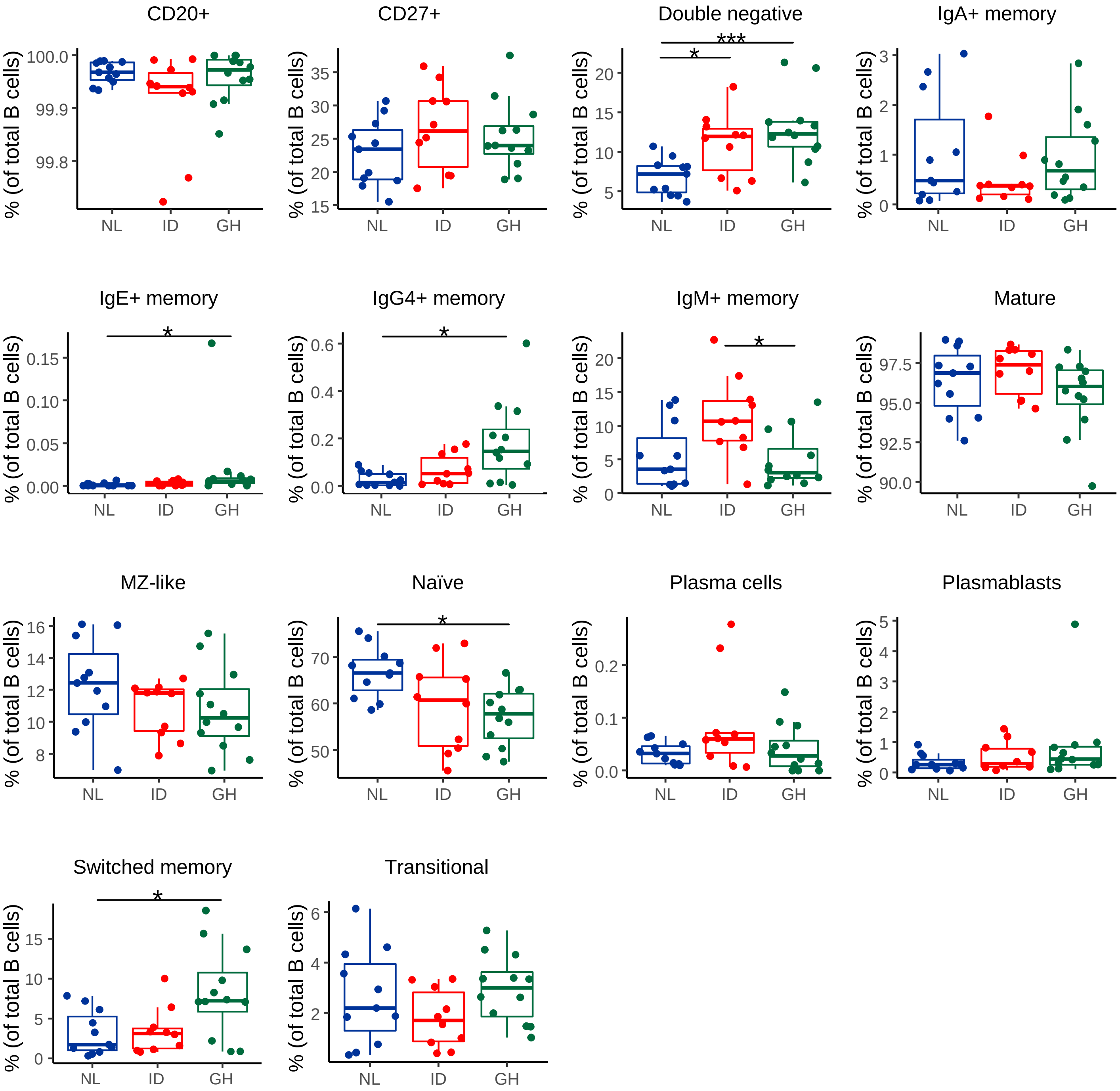

Supplementary Figure 1C:

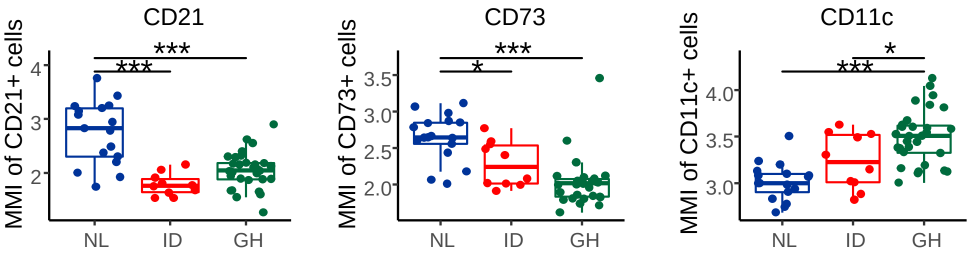

Supplementary Figure 1D:

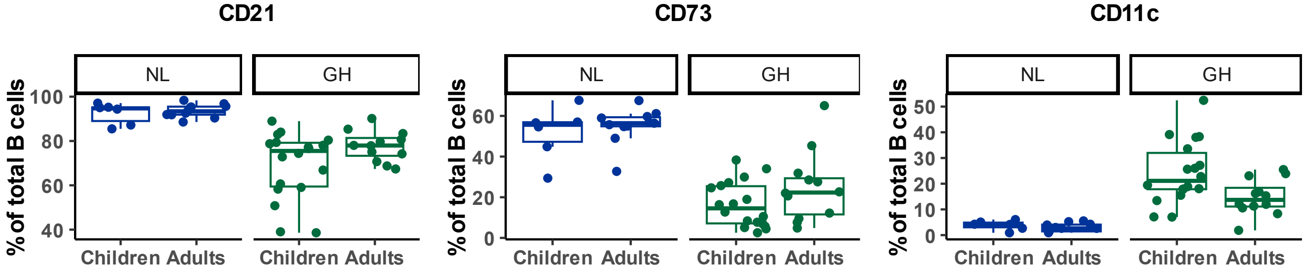

Supplementary Figure 1E:

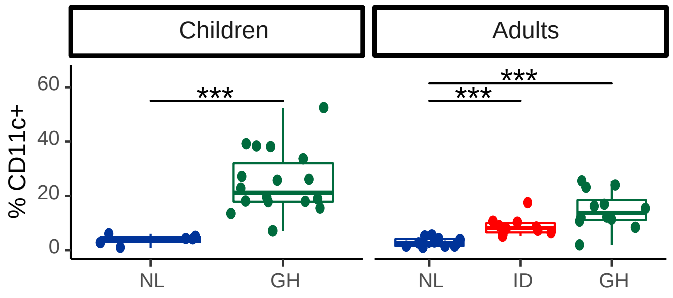

Supplementary Figure 2A:

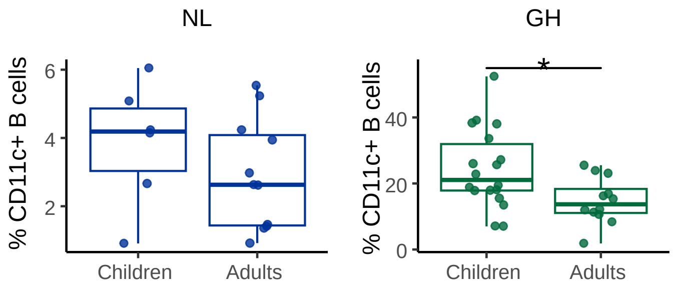

Supplementary Figure 2B:

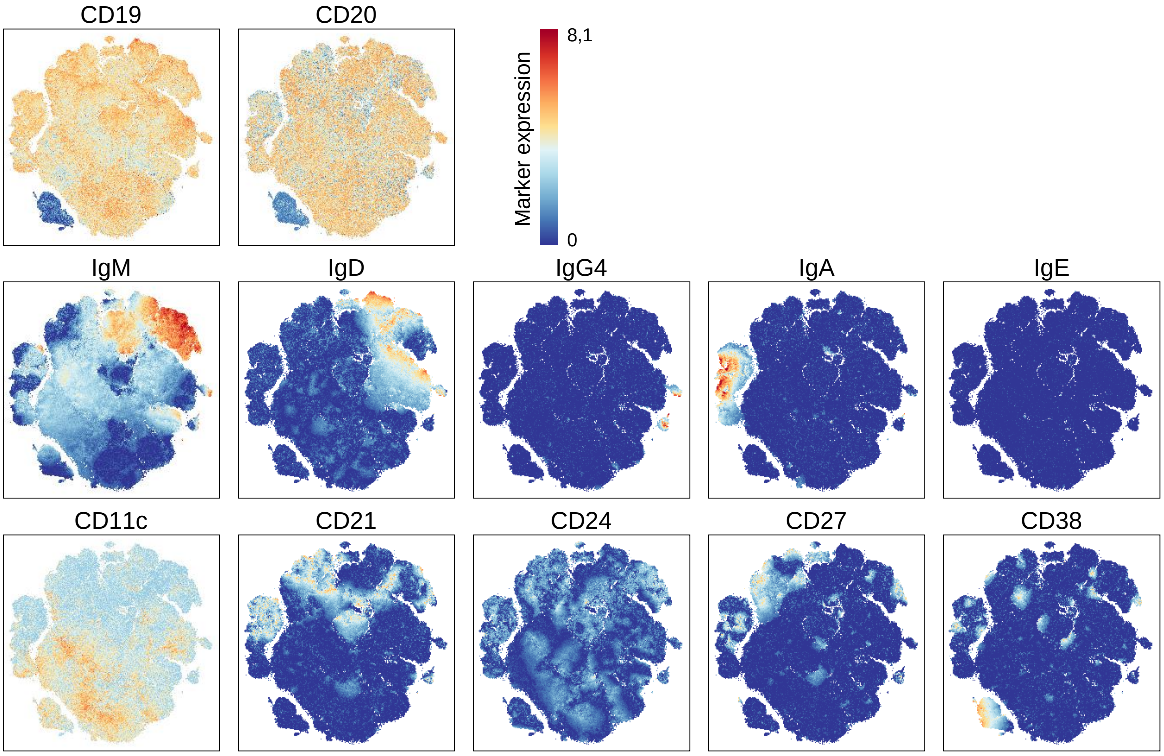

Supplementary Figure 2C:

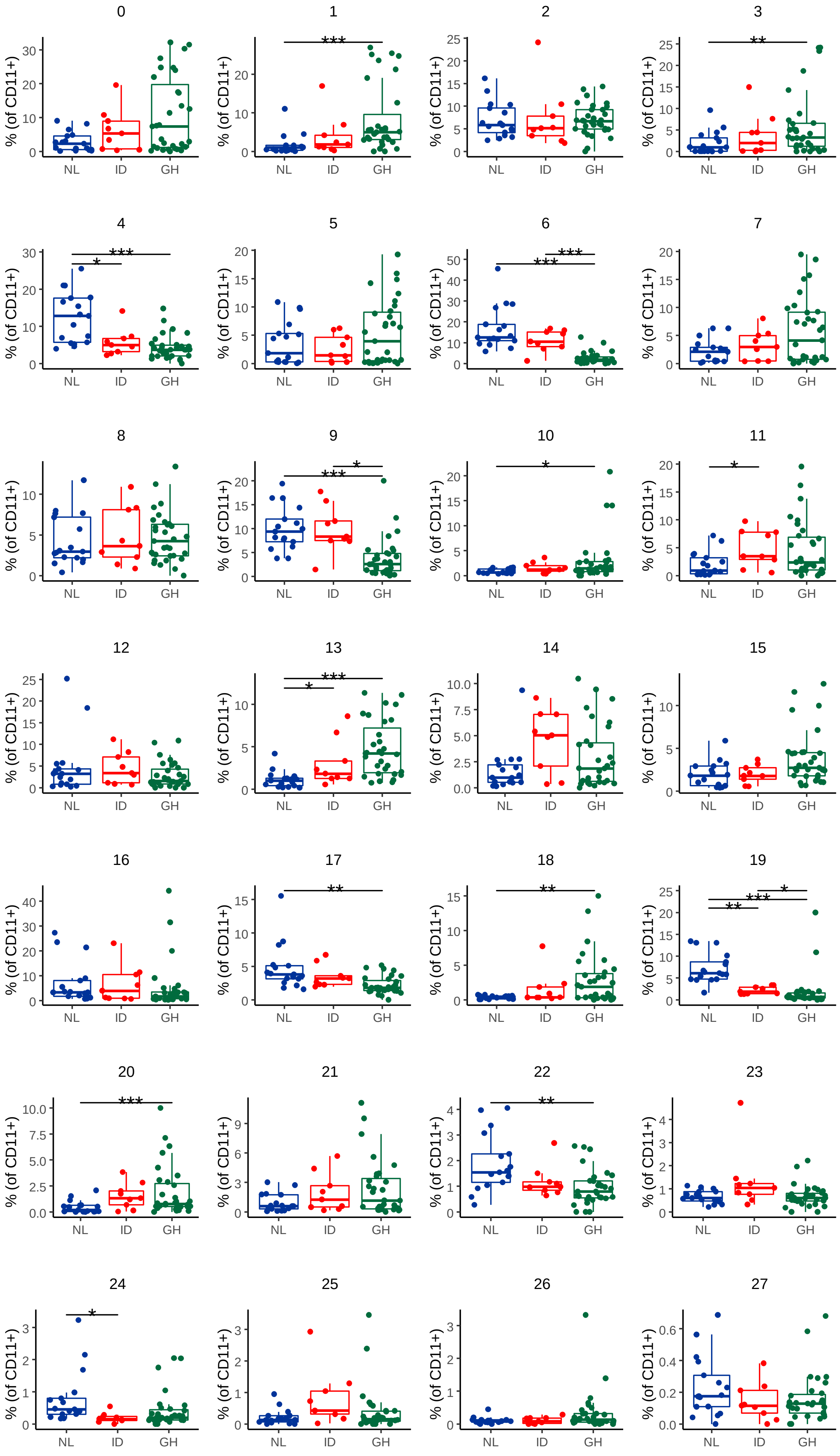

Supplementary Figure 2D:

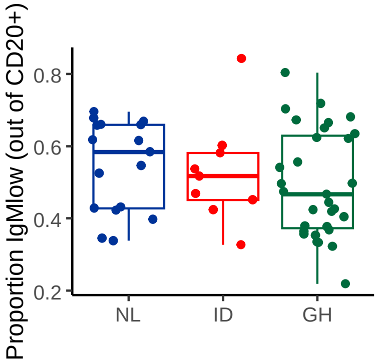

Supplementary Figure 2E:

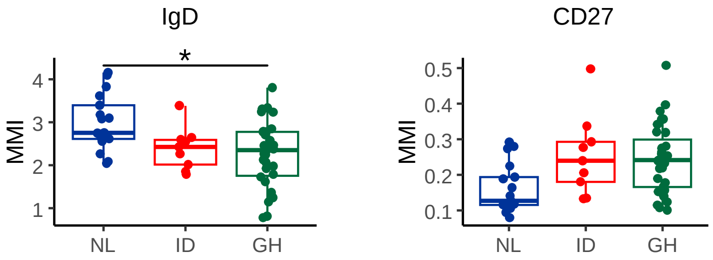

Supplementary Figure 2F:

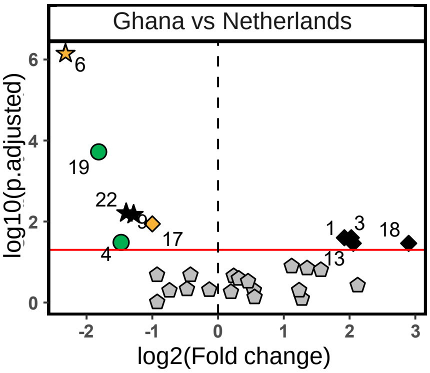

Supplementary Figure 2G:

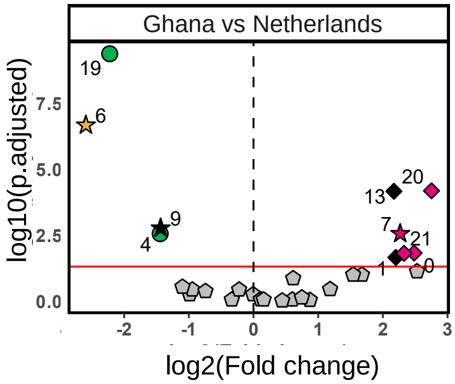

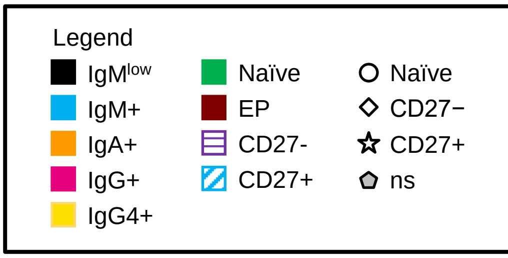

Supplementary Figure 2H:

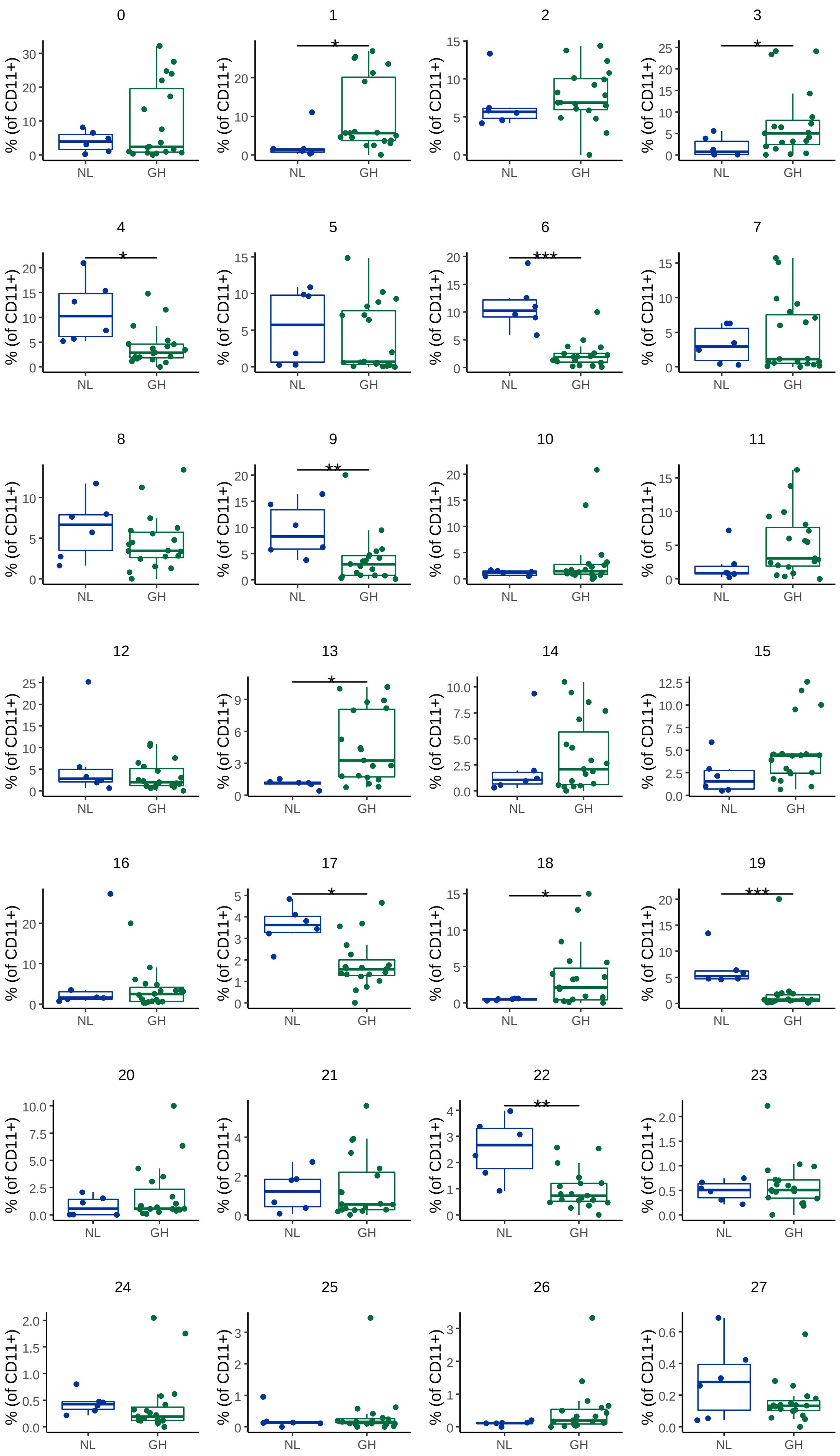

Supplementary Figure 2I:

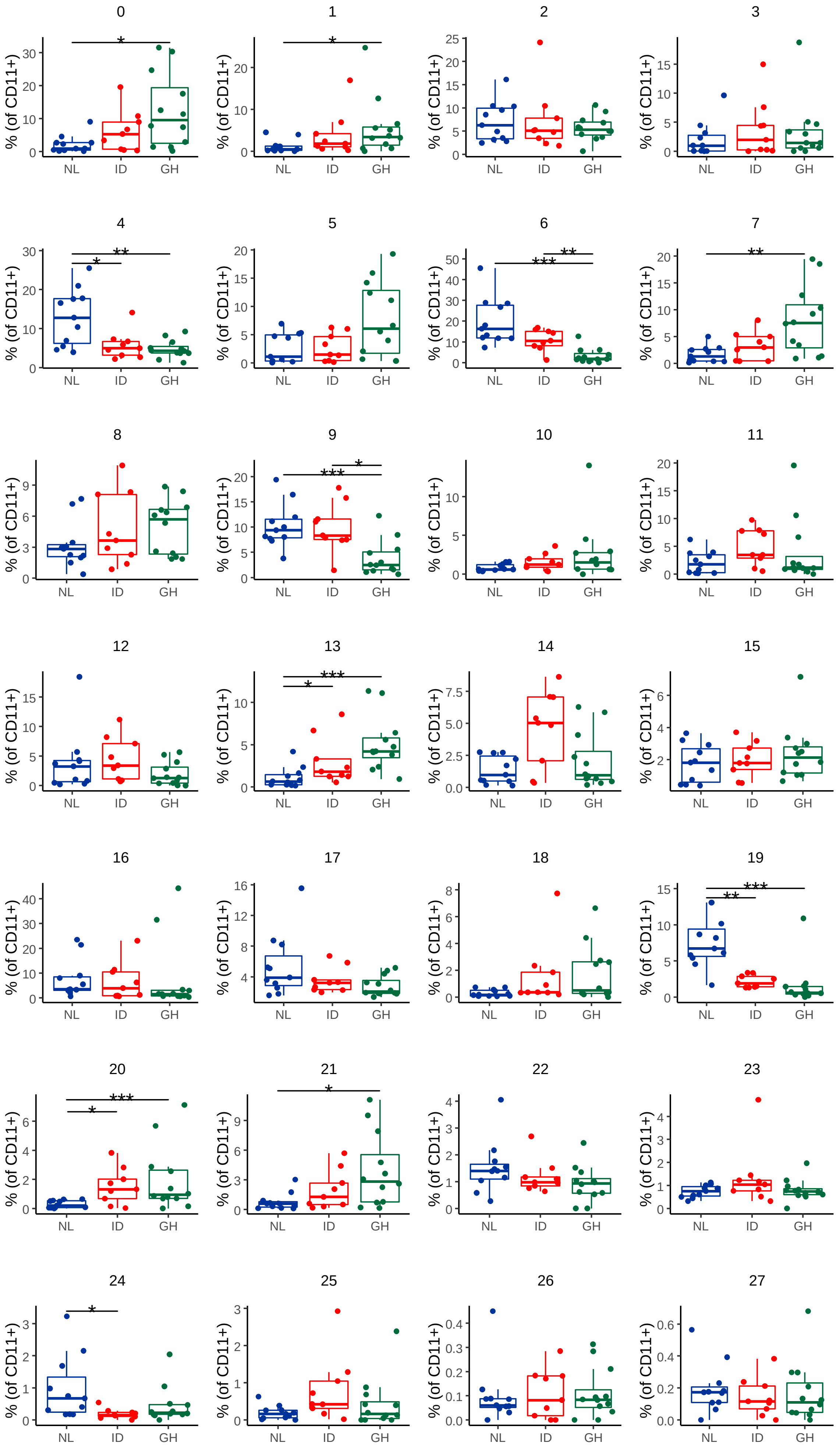

Supplementary Figure 2J:

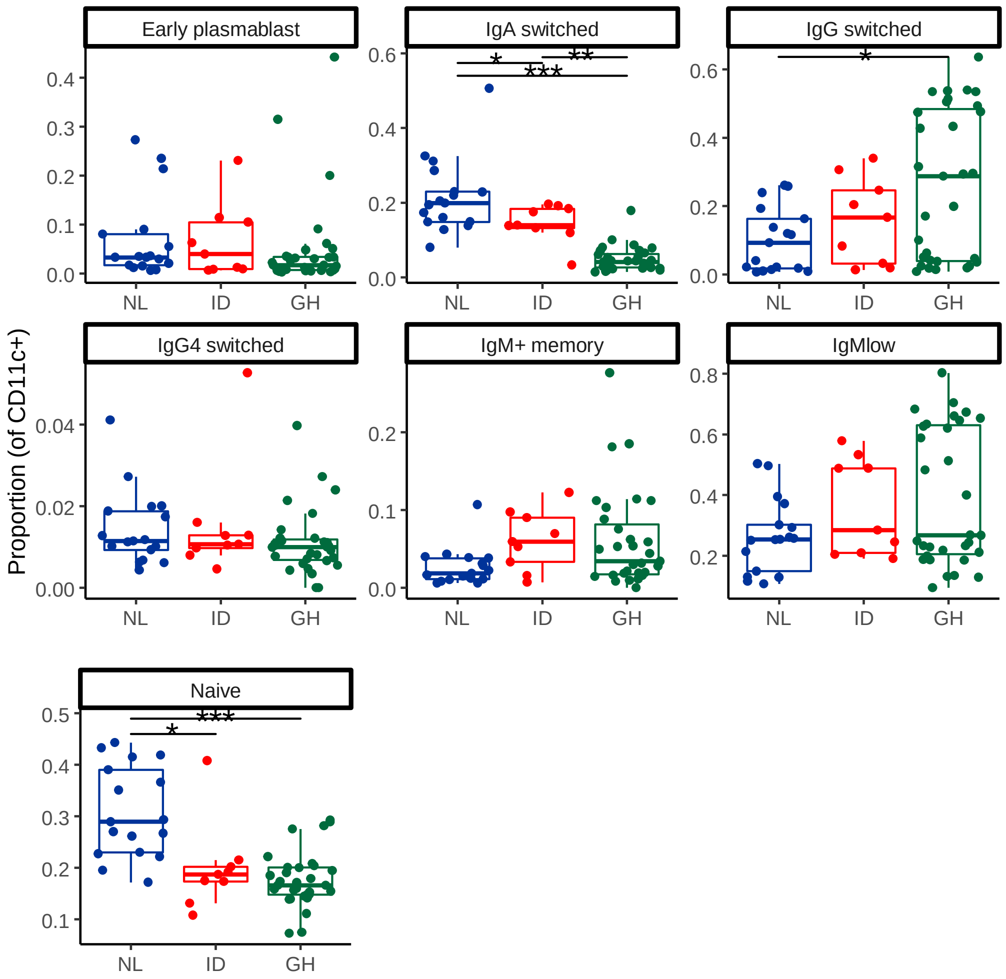

Supplementary Figure 2K:

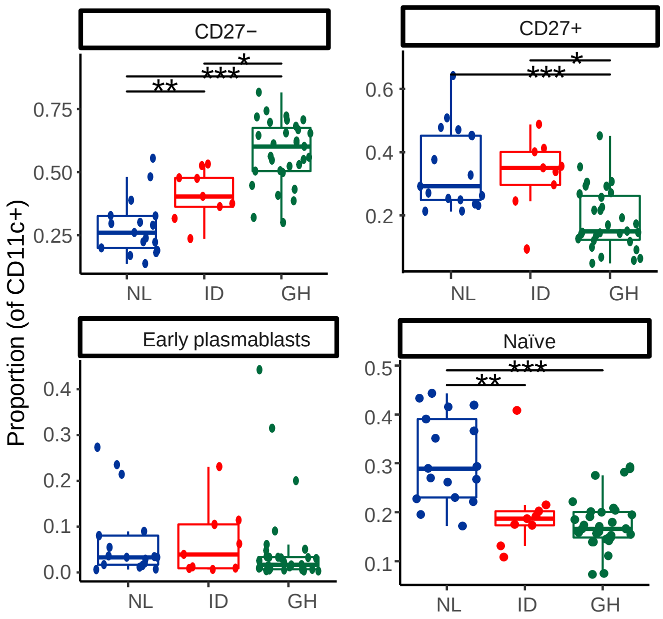

Supplementary Figure 2L:

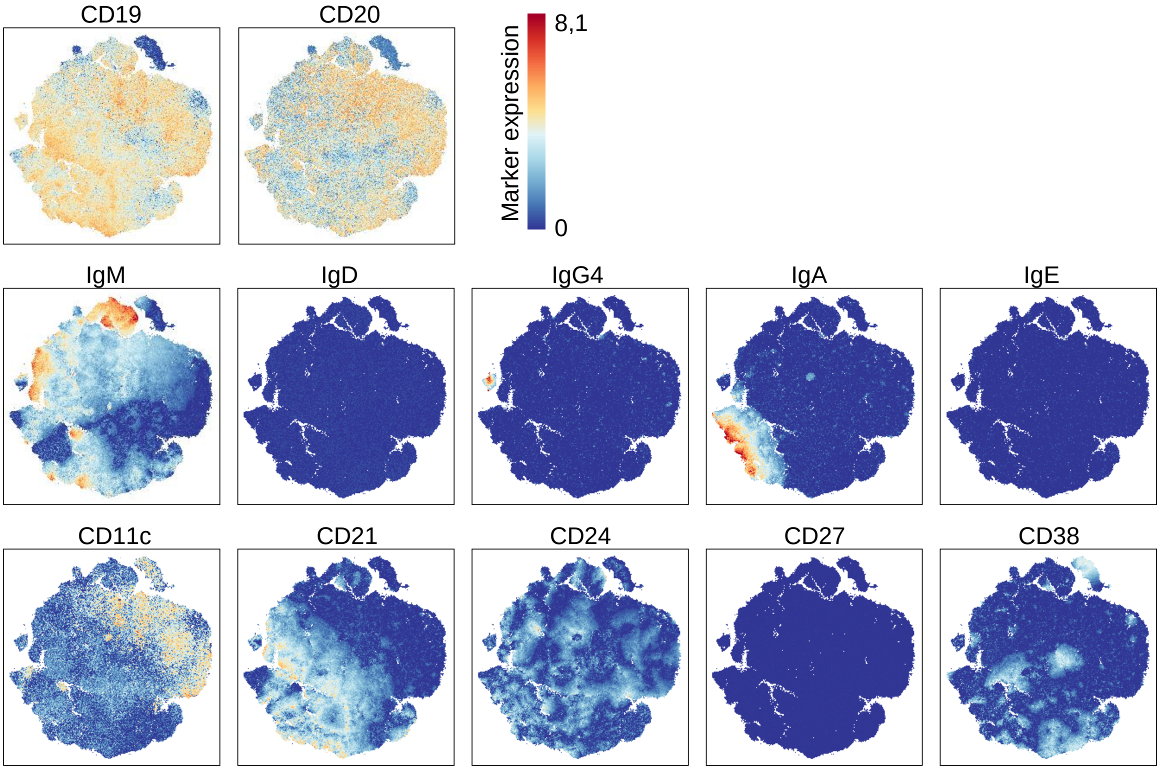

Supplementary Figure 3A:

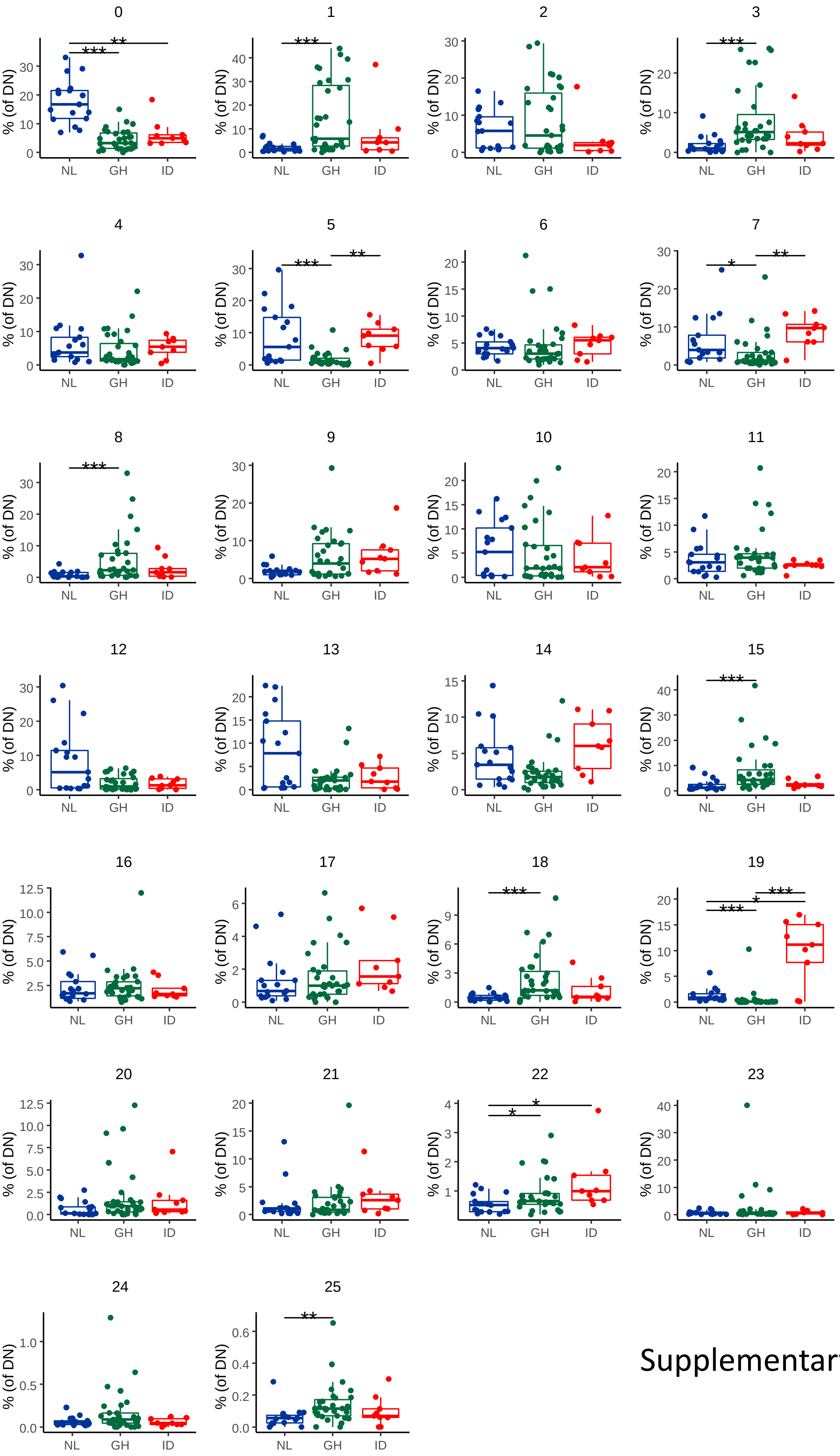

Supplementary Figure 3B:

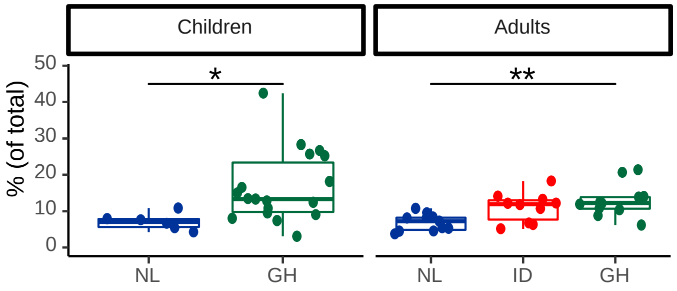

Supplementary Figure 3C:

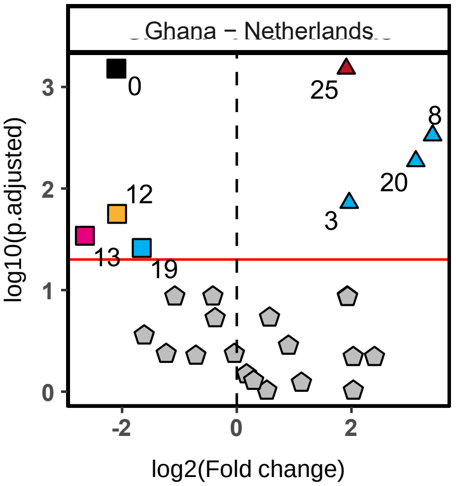

Supplementary Figure 3D:

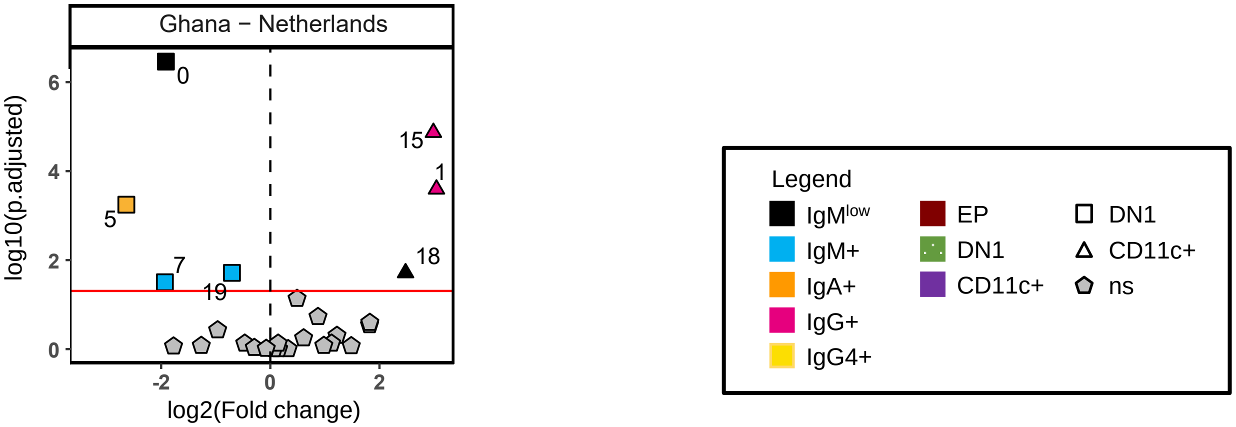

Supplementary Figure 3E:

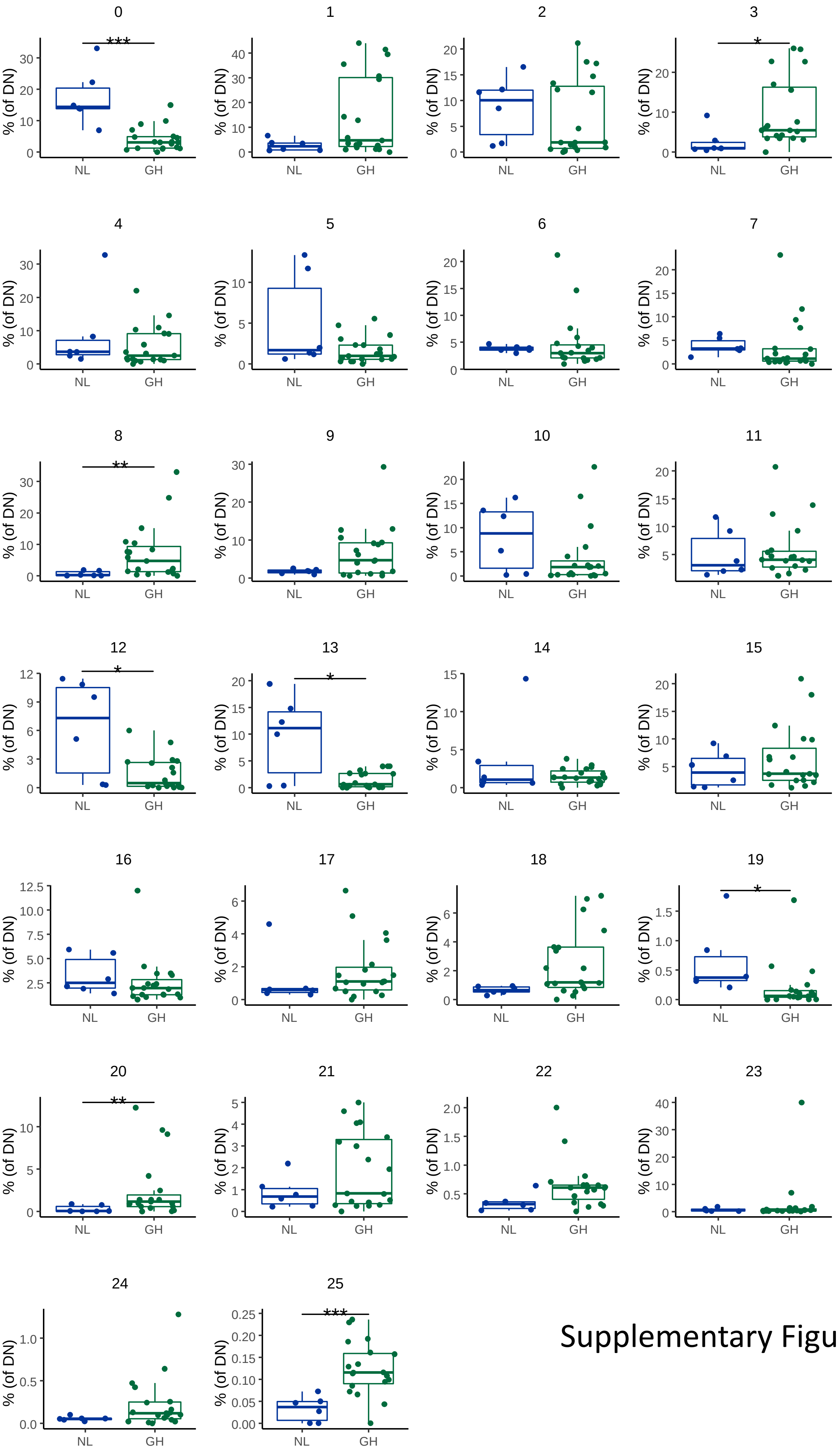

Supplementary Figure 3F:

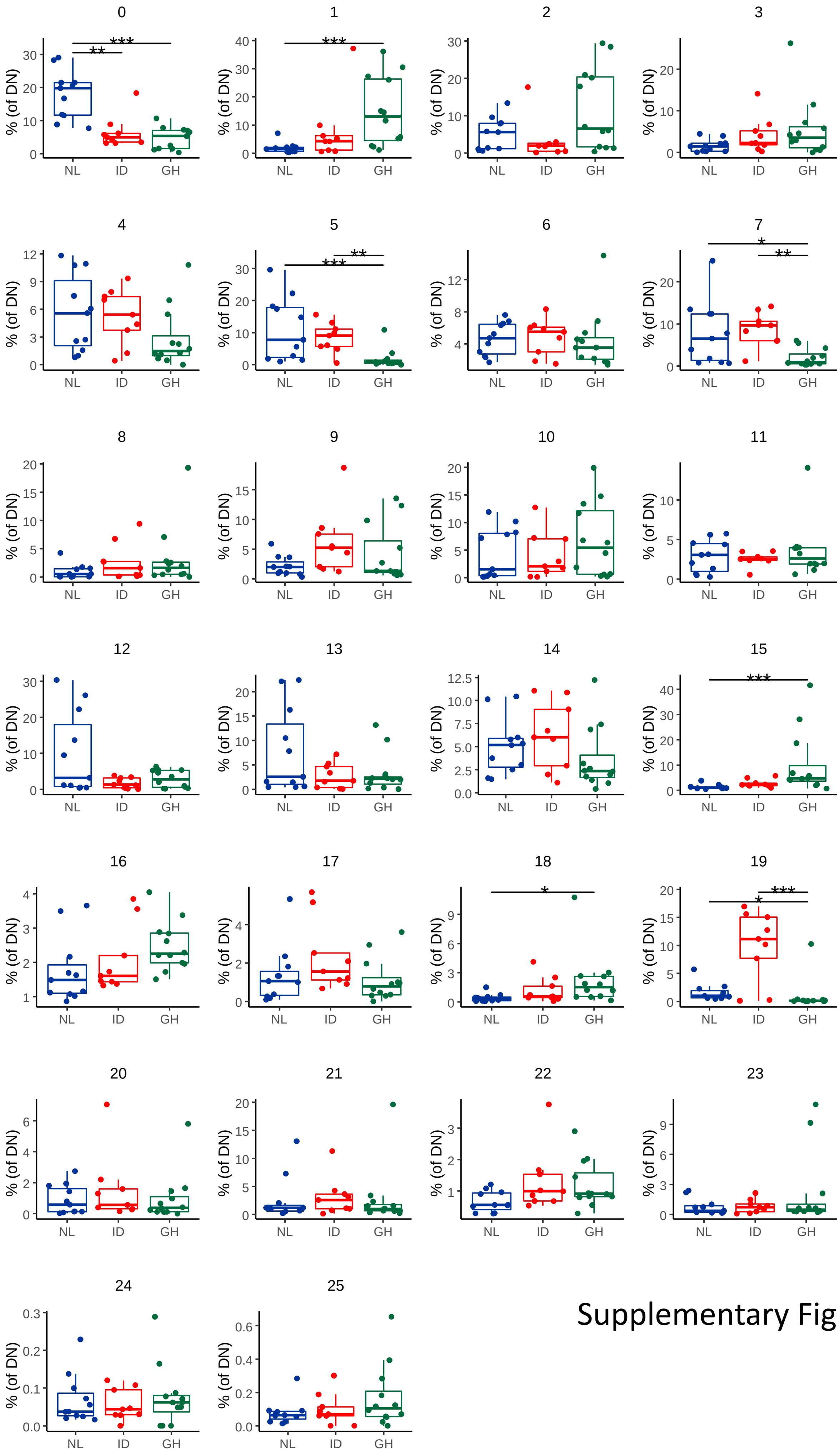

Supplementary Figure 3G:

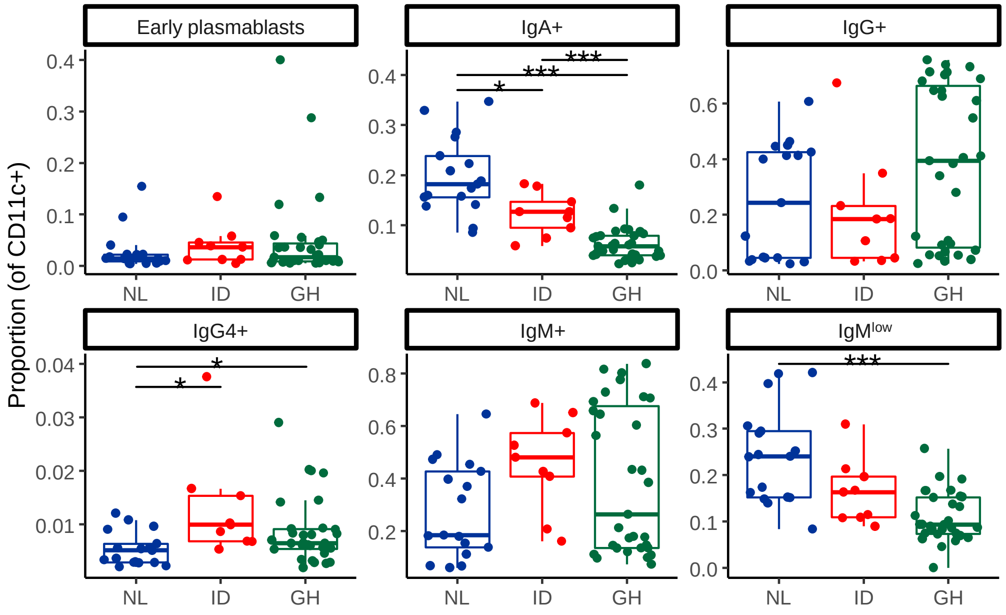

Supplementary Figure 3H:

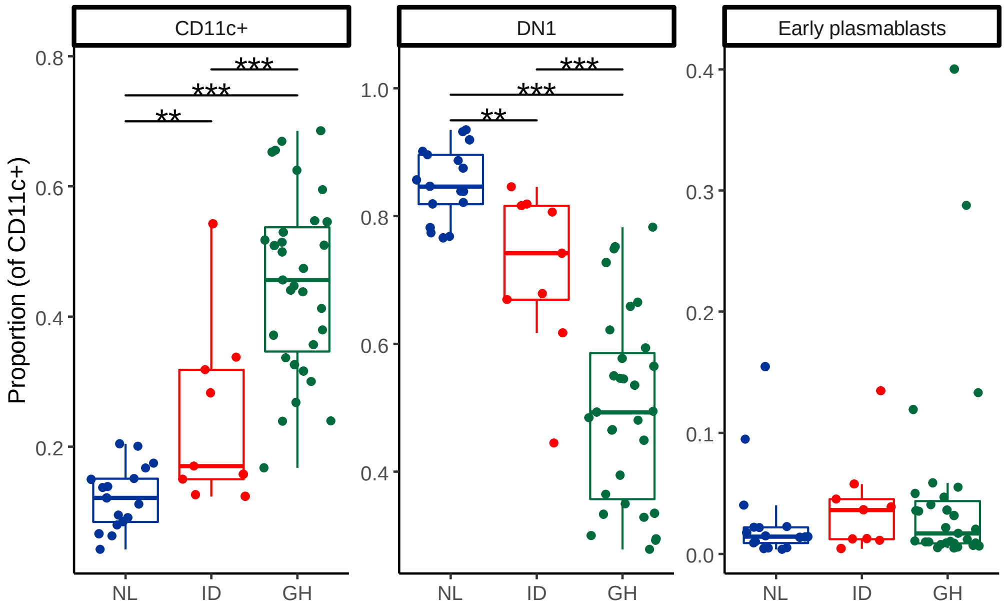

Supplementary Figure 3I:

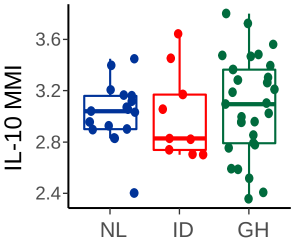

Supplementary Figure 4A:

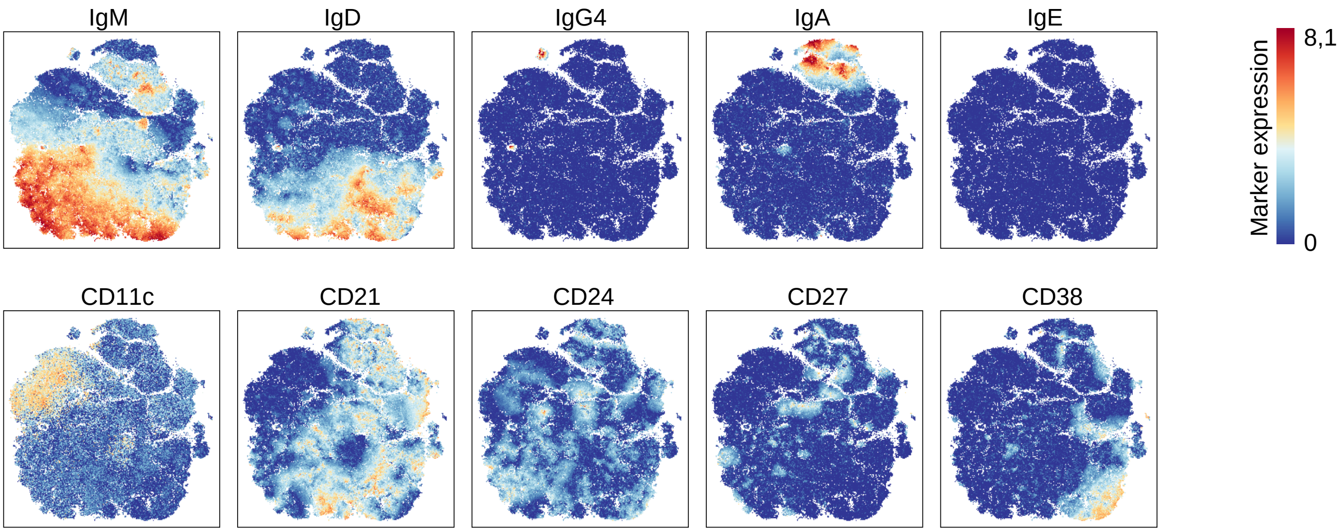

Supplementary Figure 4B:

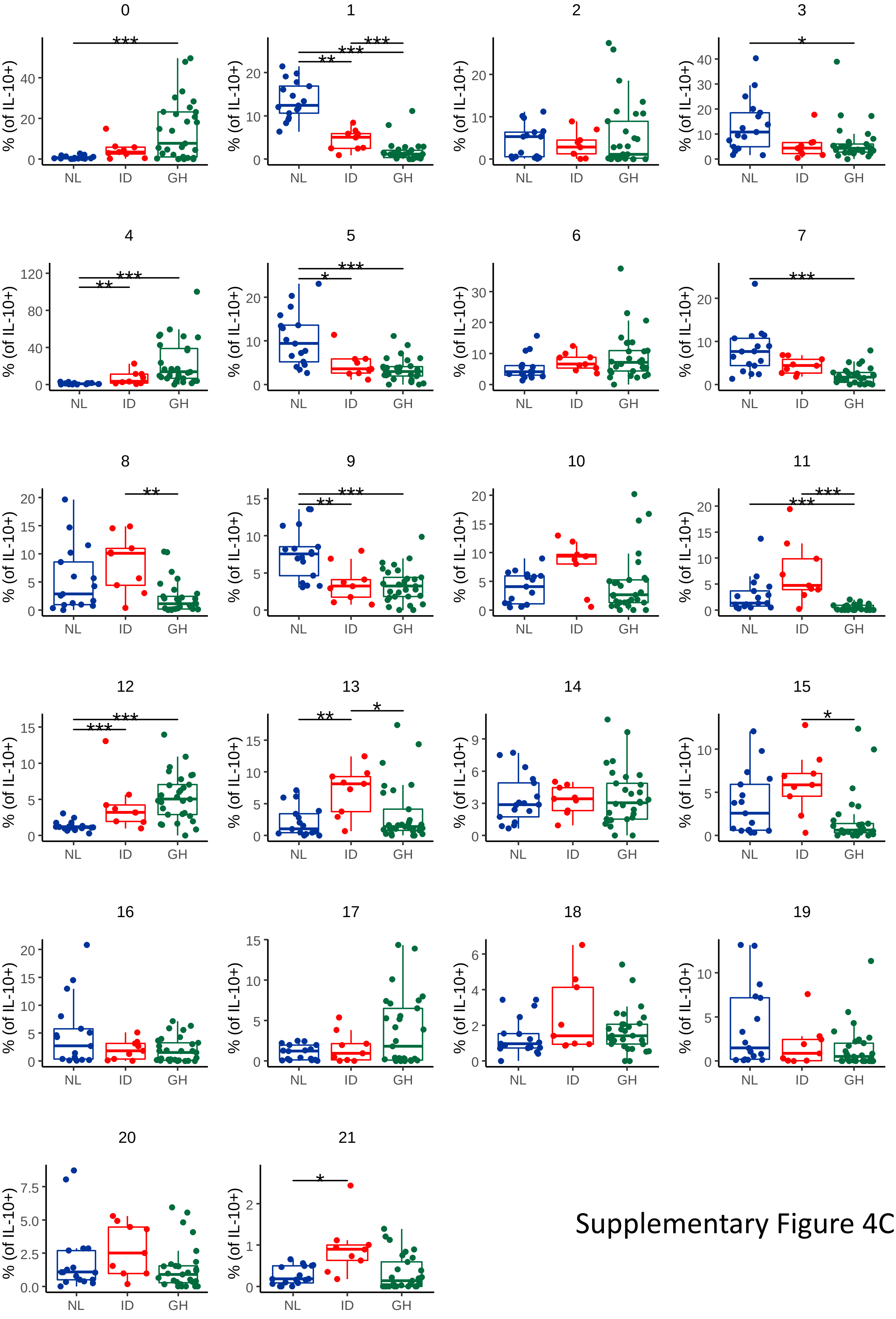

Supplementary Figure 4C:

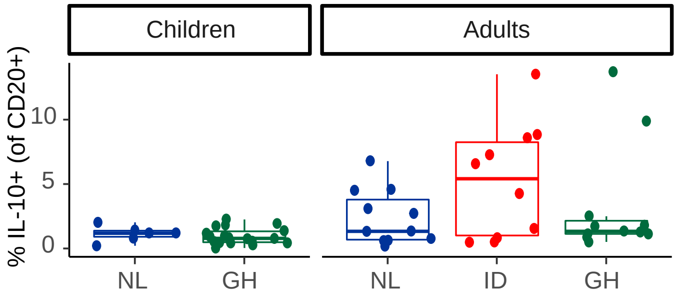

Supplementary Figure 4D:

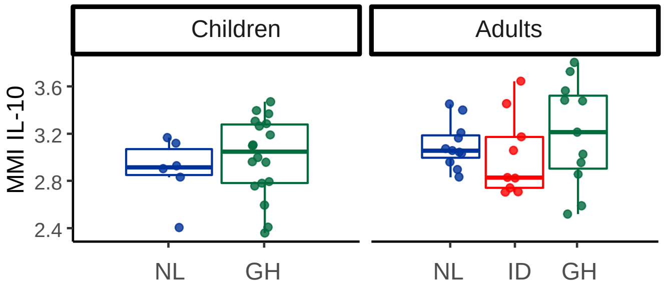

Supplementary Figure 4E:

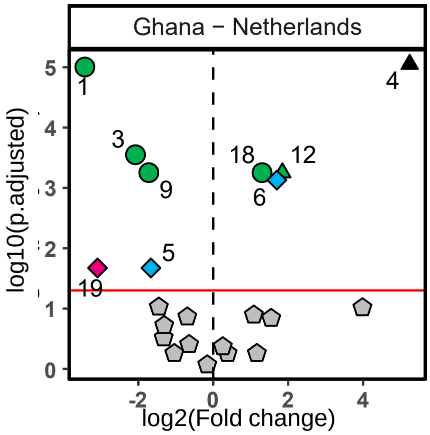

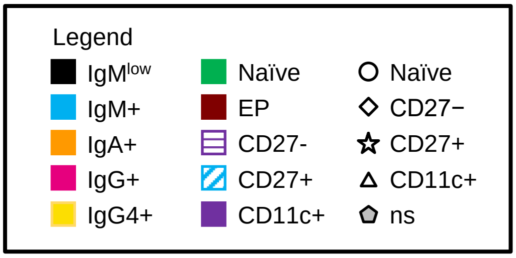

Supplementary Figure 4F:

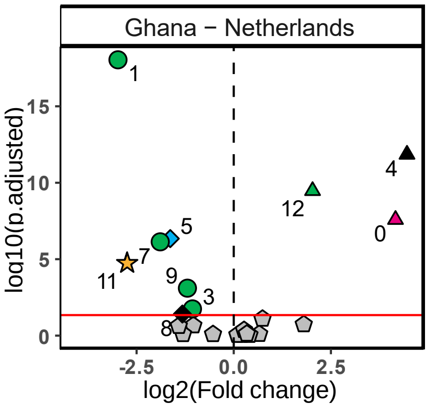

Supplementary Figure 4G:

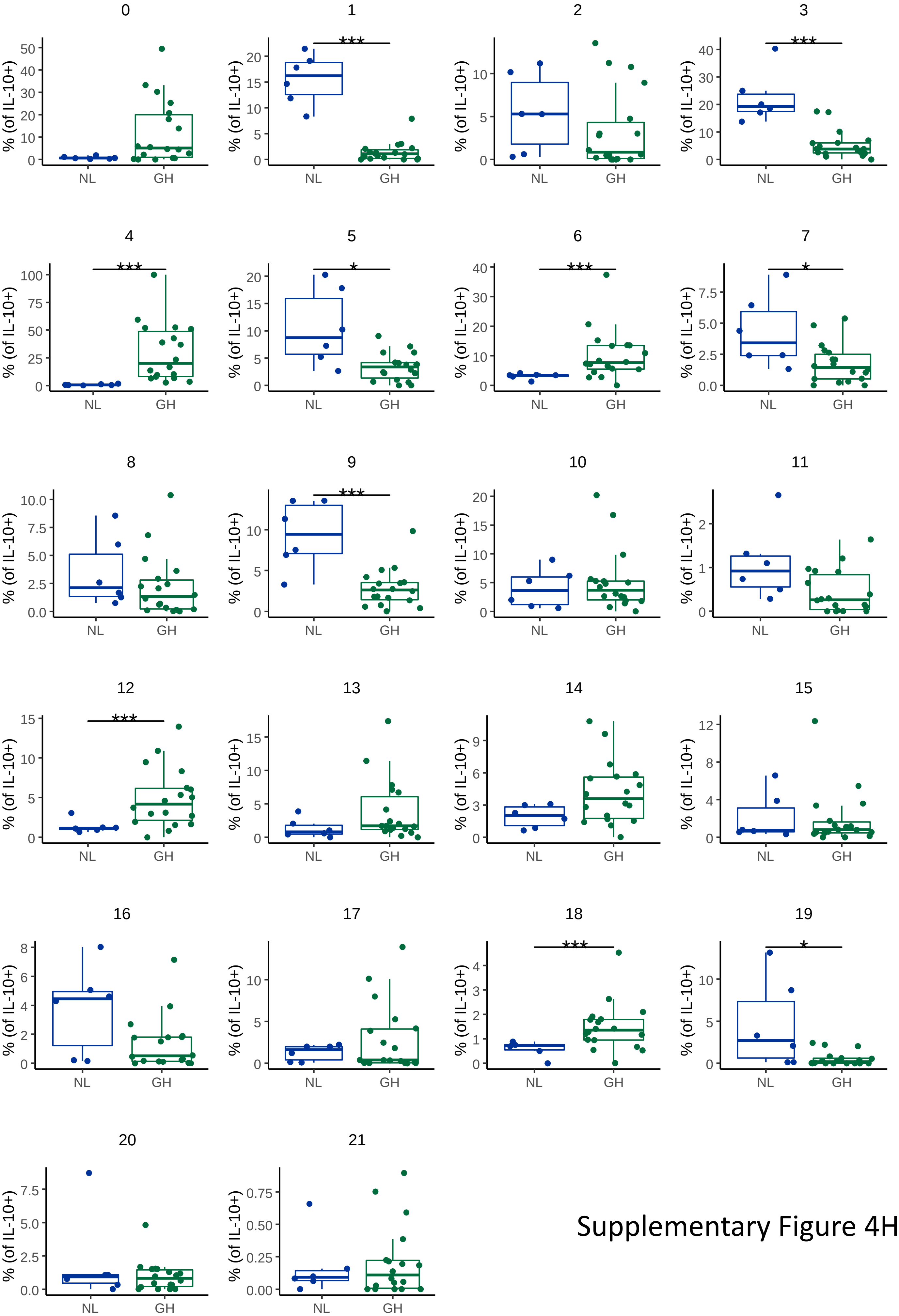

Supplementary Figure 4H:

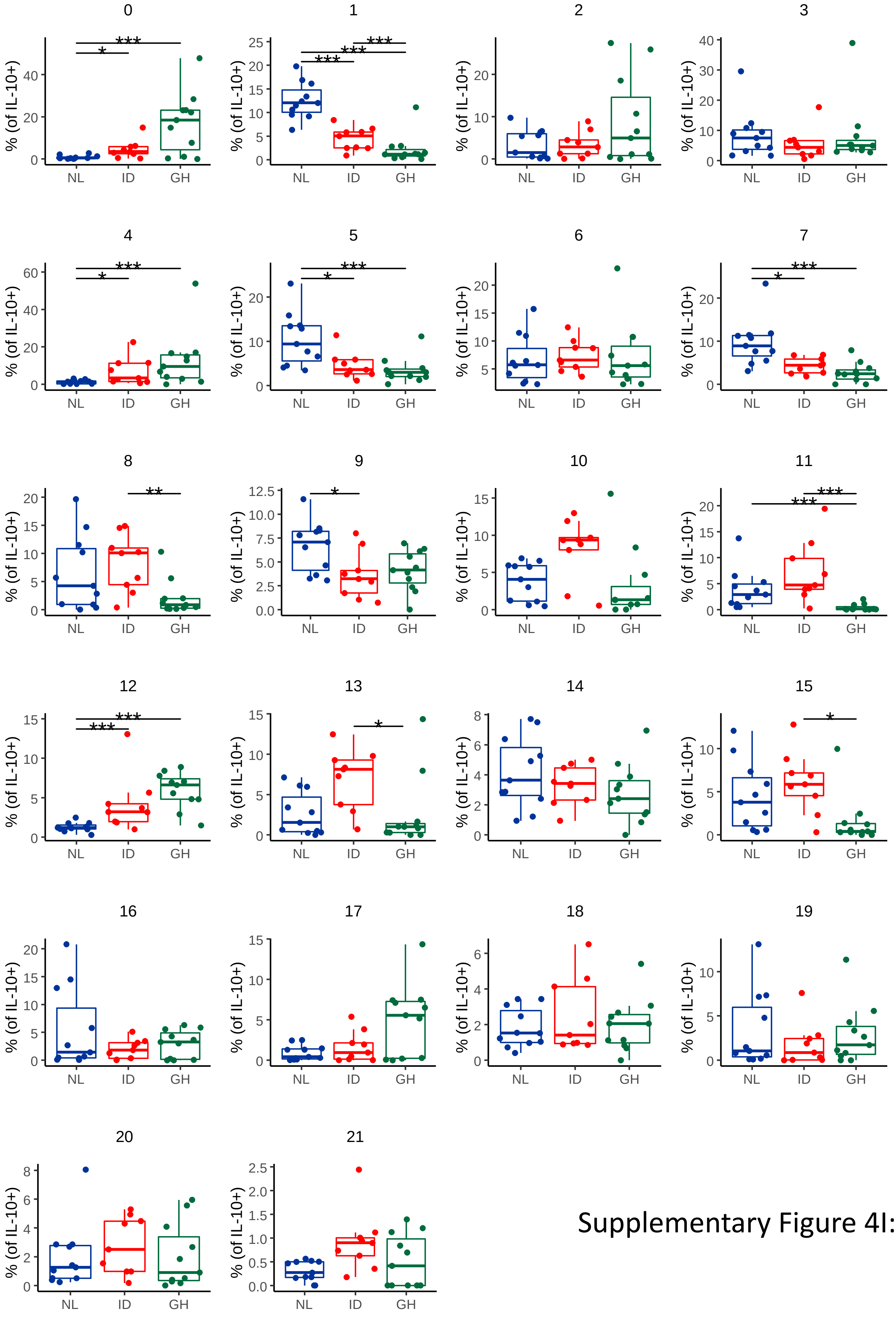

Supplementary Figure 4I:

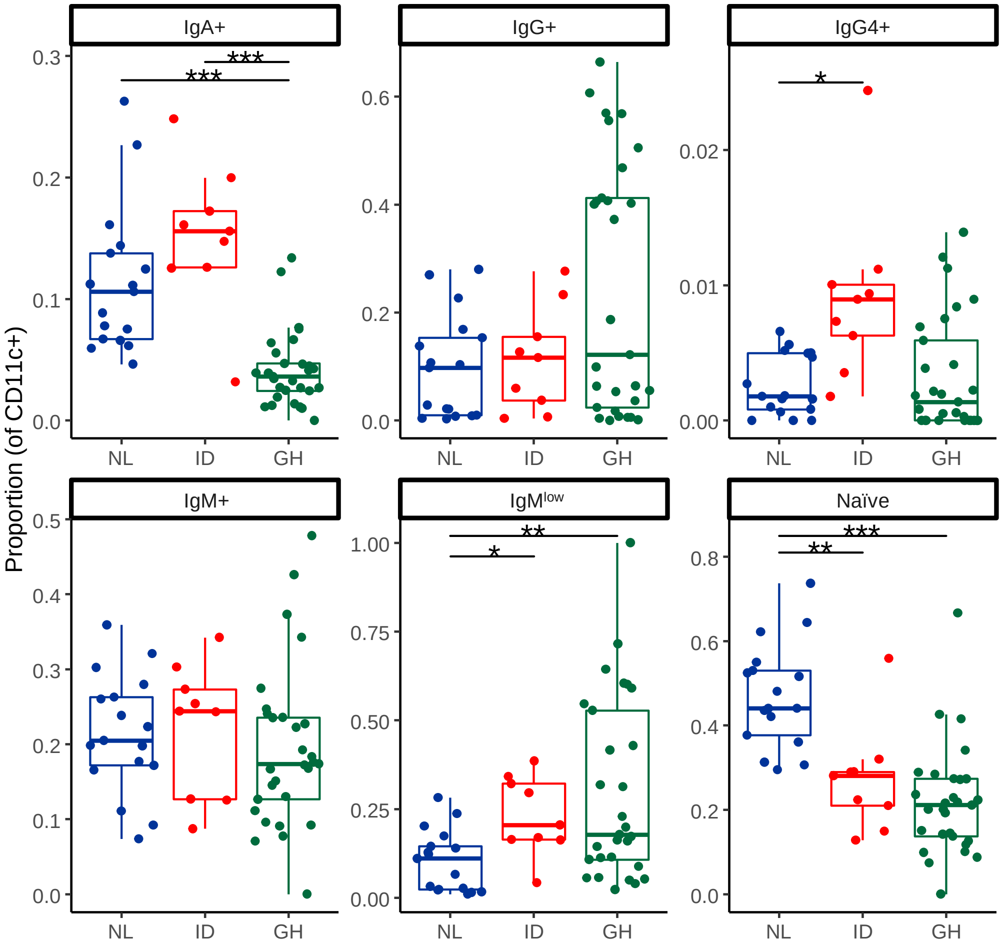

Supplementary Figure 4J:

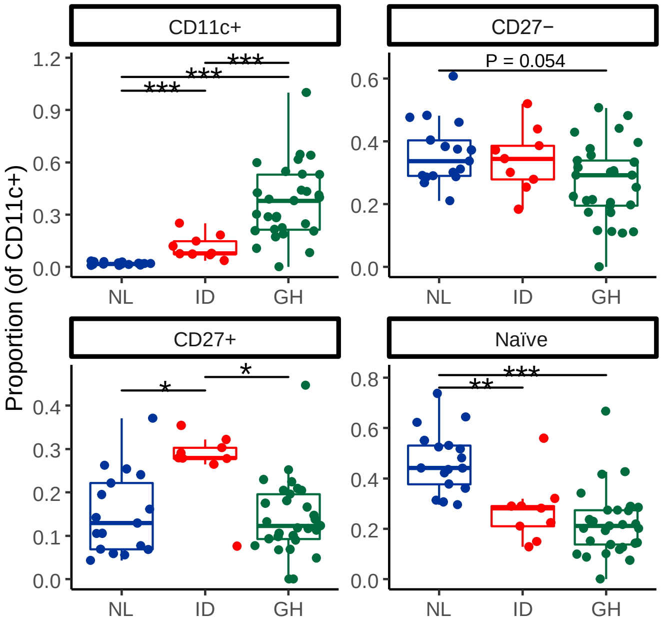

Supplementary Figure 4K:

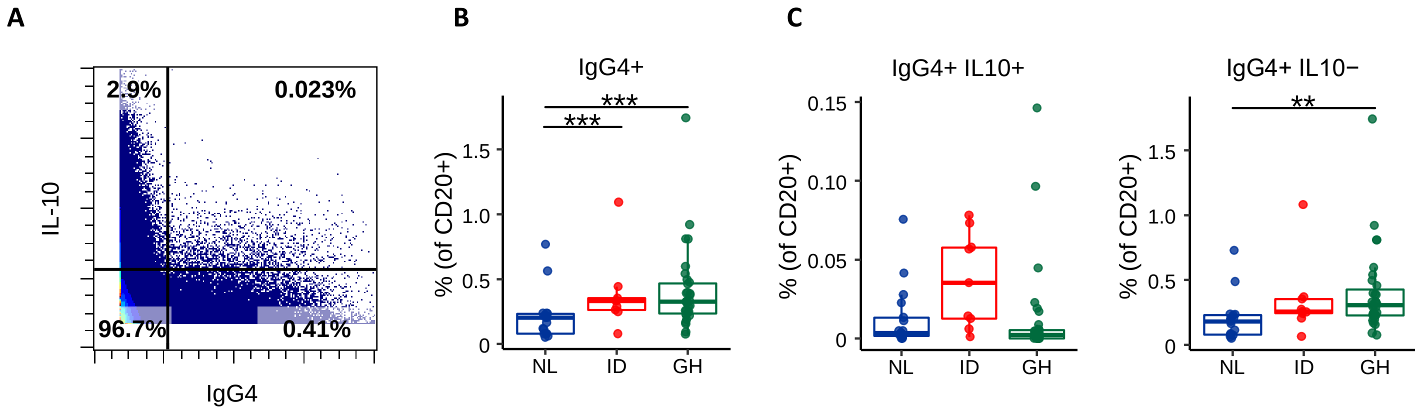

Supplementary Figure 5:

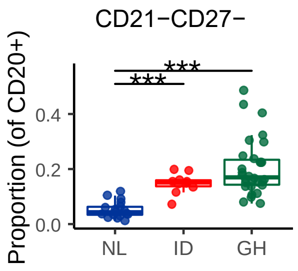

Supplementary Figure 6:

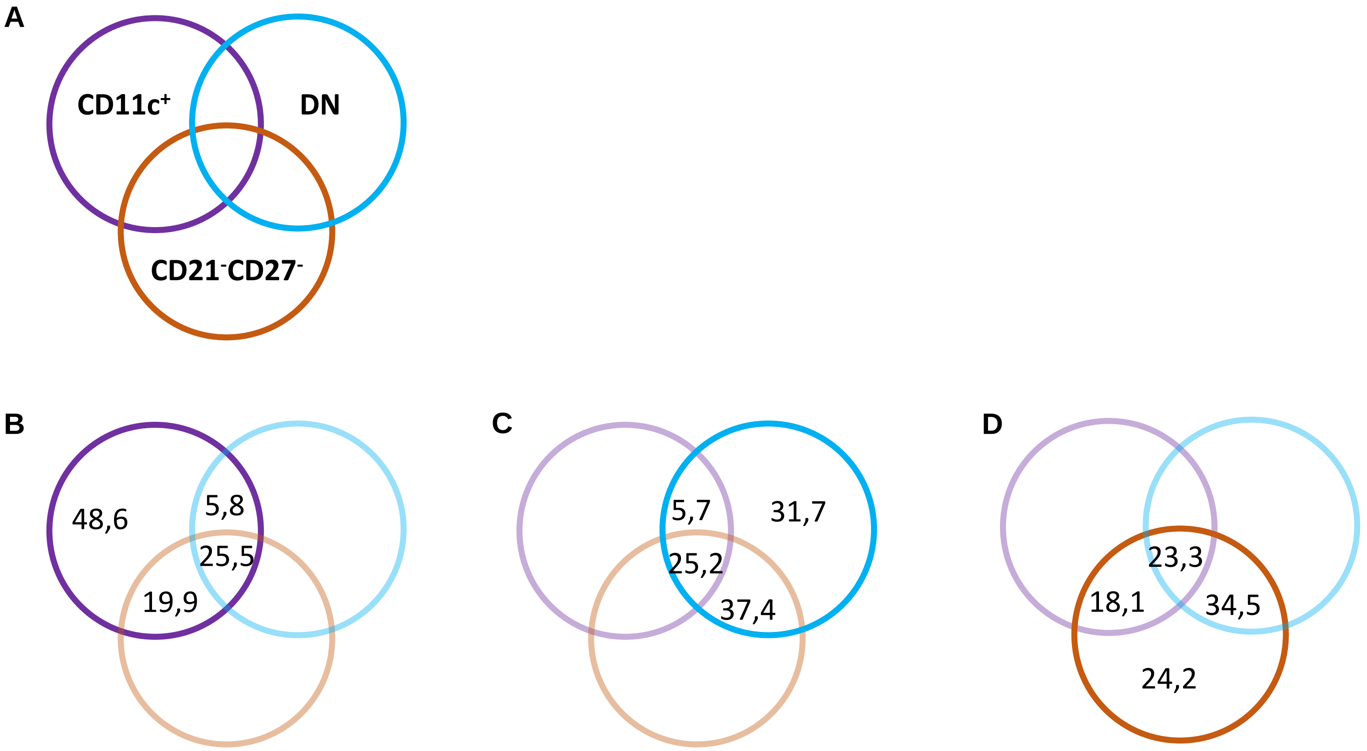

Supplementary Figure 7:

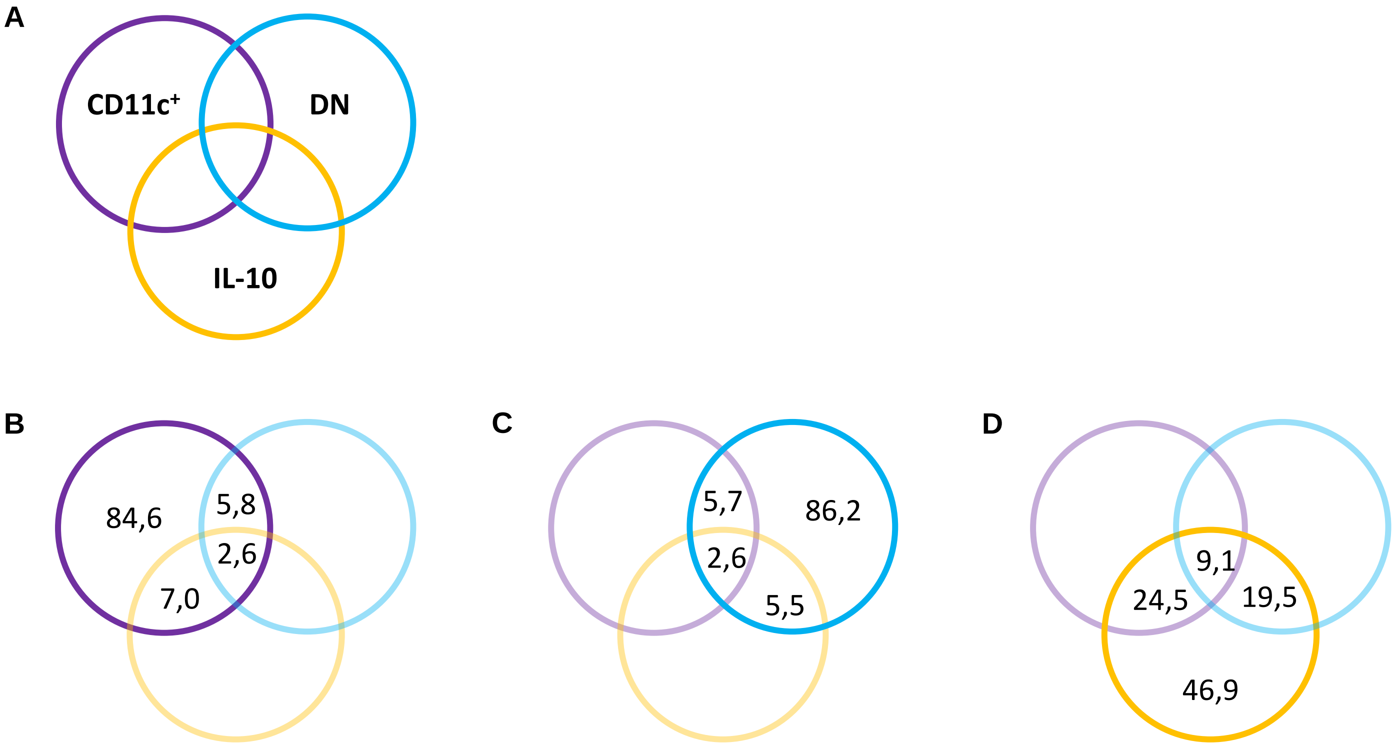

Supplementary Figure 8:

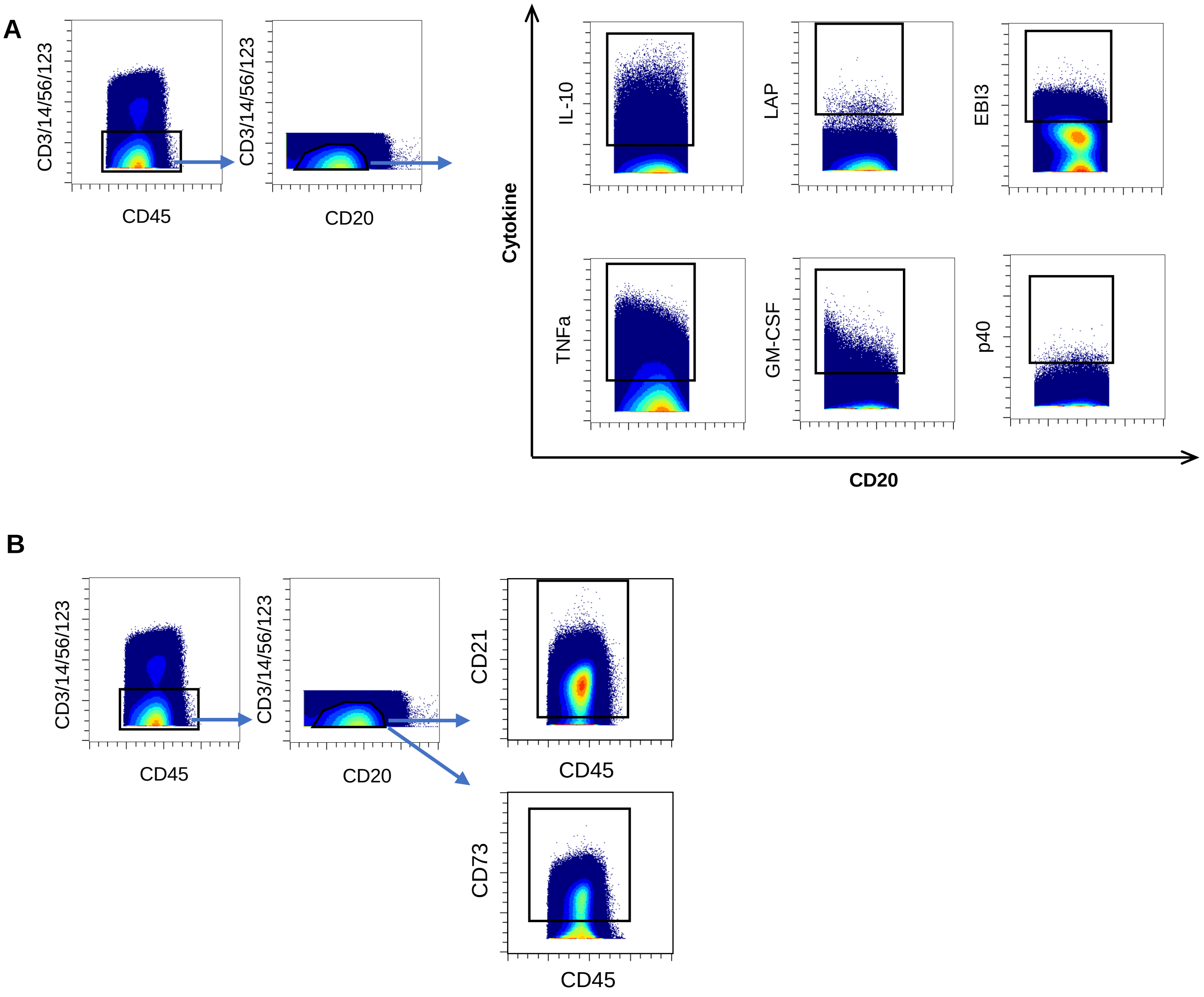

Supplementary Figure 9:
