# Supplementary material for: Multi-dimensional analysis of B cells reveals the expansion of memory and regulatory B-cell clusters in humans living in rural tropical areas
Source: Clin Exp Immunol. 2024 Aug 12;219(1):uxae074. doi: 10.1093/cei/uxae074 (PMC11771192; doi:10.1093/cei/uxae074)
Supplement: uxae074_suppl_Supplementary_Data [file uxae074_suppl_Supplementary_Data.docx]

**Supplementary Figure 1: Distinct B cell profiles between Dutch, Indonesian and Ghanaian populations.**

**(A)** Boxplots of the frequencies of various B cell populations relative to total B cells between Dutch (NL), Indonesian (ID) and Ghanaian (GH) individuals.

**(B)** Boxplots of the frequencies of various B cell populations relative to total B cells between Dutch and Ghanaian children.

**(C)** Boxplots of the frequencies of various B cell populations relative to total B cells between Dutch, Indonesian and Ghanaian adults.

**(D)** Boxplots of the mean metal intensity (MMI) of CD21 in CD21+ B cells, CD73 in CD73+ B cells and CD11c in CD11c+ B cells, compared between countries.

**(E)** Boxplots of the frequencies of CD21+ B cells, CD73+ B cells and CD11c+ B cells relative to total B cells between Dutch and Ghanaian children.

Statistics were calculated using a binomial generalized linear mixed model, * p<0.05, ** p<0.01, *** p<0.001. Total n=57, Netherlands n=17, Ghana n=30, Indonesia n=10. Total children n=24, Netherlands children n=6, Ghana children n=18. Total adults n=33, Netherlands adults n=11, Indonesian adults n=10, Ghana adults n=12.

**Supplementary Figure 2:**

**(A)** Boxplots comparing the frequencies of CD11c+ B cells in children and adults between Dutch (NL), Indonesian (ID) and Ghanaian (GH) individuals.

**(B)** Boxplots comparing the frequencies of CD11c+ B cells between children and adults in the Netherlands and Ghana.

**(C)** Optsne of the main markers used to identify different B cell populations in CD11c+ B cells.

**(D)** Boxplots comparing the frequencies of CD11c+ B cell clusters, obtained using PARC clustering, between countries for all samples.

**(E)** Boxplots comparing the frequencies of total IgM^low^ B cell, obtained by gating on CD20+ B cells, between countries for all samples.

**(F)** Boxplots of the mean metal intensity (MMI) of IgD and CD27 in IgM^low^ B cells, compared between countries.

**(G)** Volcano plots comparing the abundance of CD11c^+^ B cell clusters between Dutch and Ghanaian children. The log2(fold change) indicates the mean expression for each cluster and is plotted against log10(adjusted p value). Clusters were color coded according to several main B cell populations: naïve B cells (green), early plasmablasts (EP, dark red), IgMlow B cells (black), IgM+ (blue) memory B (MB) cells, IgA+ (orange), IgG+ (pink) or IgG4+ (yellow) switched MB cells. In addition, clusters were separated between naïve (circle), CD27- (diamond) and CD27+ (star) cells. Non significatively different clusters are in light grey pentagons. For each volcano plot comparing Ghana vs the Netherlands, clusters on the right side of the plot are higher in Ghana compared to the Netherlands, and clusters on the left side are lower in Ghana compared to the Netherlands.

**(H)** Volcano plots comparing the abundance of CD11c^+^ B cell clusters between Dutch and Ghanaian adults. Clusters were being color- and shape-coded similarly to (D).

**(I)** Boxplots comparing the frequencies of CD11c+ B cell clusters between Dutch and Ghanaian children.

**(J)** Boxplots comparing the frequencies of CD11c+ B cell clusters between Dutch, Indonesian and Ghanaian adults.

**(K)** CD11c+ B cell clusters were sorted into different B cell populations as defined in (D), namely naïve B cells, early plasmablasts, IgMlow B cells, IgM+ MB cells, IgA+, IgG+ or IgG4+ switched MB cells. The frequency of these populations was then plotted in boxplots and compared between countries.

**(L)** CD11c+ B cell clusters were separated into naïve, CD27-, CD27+ B cells and early plasmablasts, as defined in (D). The frequency of these populations was then plotted in boxplots and compared between countries.

Statistics were calculated using a binomial generalized linear mixed model, * p<0.05, ** p<0.01, *** p<0.001. Total n=57, Netherlands n=17, Ghana n=30, Indonesia n=10. Total children n=24, Netherlands children n=6, Ghana children n=18. Total adults n=33, Netherlands adults n=11, Indonesian adults n=10, Ghana adults n=12.

**Supplementary Figure 3:**

**(A)** Optsne of the main markers used to identify different B cell populations in double negative (DN) B cells.

**(B)** Boxplots comparing the frequencies of DN B cell clusters, obtained using PARC clustering, between Dutch (NL), Indonesian (ID) and Ghanaian (GH) individuals.

**(C)** Boxplots comparing the frequency of DN B cells in children and adults between countries.

**(D)** Volcano plots comparing the abundance of DN B cell clusters between Dutch and Ghanaian children. The log2(fold change) indicates the mean expression for each cluster and is plotted against log10(adjusted p value). Clusters were color coded according to several main B cell populations: early plasmablasts (EP, dark red), IgMlow B cells (black), IgM+ (blue) memory B (MB) cells, IgA+ (orange), IgG+ (pink) or IgG4+ (yellow) switched MB cells. In addition, clusters were separated between DN1 (square) and CD11c+ DN2 (triangle) cells. Non significatively different clusters are in light grey pentagons. For each volcano plot comparing Ghana vs the Netherlands, clusters on the right side of the plot are higher in Ghana compared to the Netherlands, and clusters on the left side are lower in Ghana compared to the Netherlands.

**(E)** Volcano plots comparing the abundance of DN B cell clusters between Dutch and Ghanaian adults. Clusters were color- and shape-coded similarly to (D).

**(F)** Boxplots comparing the frequencies of DN B cell clusters between Dutch and Ghanaian children.

**(G)** Boxplots comparing the frequencies of DN B cell clusters between Dutch, Indonesian and Ghanaian adults.

**(H)** DN B cell clusters were sorted into different B cell populations as defined in (D), namely early plasmablasts, IgMlow B cells, IgM+ MB cells, IgA+, IgG+ or IgG4+ switched MB cells. The frequency of these populations was then plotted in boxplots and compared between countries.

**(I)** DN B cell clusters were separated into early plasmablasts, DN1 and CD11c+ DN2, as defined in (D). The frequency of these populations was then plotted in boxplots and compared between countries.

Statistics were calculated using a binomial generalized linear mixed model, * p<0.05, ** p<0.01, *** p<0.001. Total n=57, Netherlands n=17, Ghana n=30, Indonesia n=10. Total children n=24, Netherlands children n=6, Ghana children n=18. Total adults n=33, Netherlands adults n=11, Indonesian adults n=10, Ghana adults n=12.

**Supplementary Figure 4:**

**(A)** Boxplots of the mean metal intensity (MMI) of IL-10 in IL-10+ B cells, compared between Dutch (NL), Indonesian (ID) and Ghanaian (GH) individuals.

**(B)** Optsne of the main markers used to identify different B cell populations in IL-10+ B cells.

**(C)** Boxplots comparing the frequencies of IL-10+ B cell clusters, obtained using PARC clustering, between countries.

**(D)** Boxplots comparing the frequency of IL-10+ B cells in children and adults between countries.

**(E)** Boxplots comparing the MMI of IL-10 in children and adults between countries.

**(F)** Volcano plots comparing the abundance of IL-10+ B cell clusters between Dutch and Ghanaian children. The log2(fold change) indicates the mean expression for each cluster and is plotted against log10(adjusted p value). Clusters were color coded according to several main B cell populations: naïve B cells (green), IgMlow B cells (black), IgM+ (blue) memory B (MB) cells, IgA+ (orange), IgG+ (pink) or IgG4+ (yellow) switched MB cells. In addition, clusters were separated between naïve (round), CD27- (diamond), CD27+ (star) and CD11c+ (triangle) B cells. Non significatively different clusters are in light grey pentagons. For each volcano plot comparing Ghana vs the Netherlands, clusters on the right side of the plot are higher in Ghana compared to the Netherlands, and clusters on the left side are lower in Ghana compared to the Netherlands.

**(G)** Volcano plots comparing the abundance of DN B cell clusters between Dutch and Ghanaian adults. Clusters were color- and shape-coded similarly to (F).

**(H)** Boxplots comparing the frequencies of IL-10+ B cell clusters between Dutch and Ghanaian children.

**(I)** Boxplots comparing the frequencies of IL-10+ B cell clusters between Dutch, Indonesian and Ghanaian adults.

**(J)** IL-10+ B cell clusters were sorted into different B cell populations as defined in (F), namely naïve B cells, IgMlow B cells, IgM+ MB cells, IgA+, IgG+ or IgG4+ switched MB cells. The frequency of these populations was then plotted in boxplots and compared between countries.

**(K)** IL-10+ B cell clusters were separated into naïve, CD27-, CD27+ and CD11c+ B cells, as defined in (F). The frequency of these populations was then plotted in boxplots and compared between countries.

Statistics were calculated using a binomial generalized linear mixed model, * p<0.05, ** p<0.01, *** p<0.001. Total n=57, Netherlands n=17, Ghana n=30, Indonesia n=10. Total children n=24, Netherlands children n=6, Ghana children n=18. Total adults n=33, Netherlands adults n=11, Indonesian adults n=10, Ghana adults n=12.

**Supplementary Figure 5: IgG4^+^ B cells are mainly IL-10^-^ B cells**

**(A)** 2D plot showing the mean percentage of IL-10+, IgG4+, IL-10+IgG4+ and IL-10-IgG4- B cells (all samples combined).

**(B)** Boxplot comparing the percentage of IgG4+ B cells between Dutch (NL), Indonesian (ID) and Ghanaian (GH) subjects.

**(C)** Boxplots comparing the frequencies of IgG4+IL-10+ and IgG4+IL-10- B cells between countries.

Statistics were calculated using a binomial generalized linear mixed model, * p<0.05, ** p<0.01, *** p<0.001. Total n=57, Netherlands n=17, Ghana n=30, Indonesia n=10.

**Supplementary Figure 6:**

Boxplot comparing the proportion of CD21^-^CD27^-^ B cells in CD20^+^ B cells between Dutch (NL), Indonesian (ID) and Ghanaian (GH) subjects.

Statistics were calculated using a binomial generalized linear mixed model, * p<0.05, ** p<0.01, *** p<0.001. Total n=57, Netherlands n=17, Ghana n=30, Indonesia n=10.

**Supplementary Figure 7: Overlap between CD11c^+^, DN and CD21^-^CD27^-^ B cell populations**

(A) Reference Venn diagram showing the position of the 3 main populations CD11c^+^ (purple circle), DN (blue circle), and CD21^-^CD27^-^ (dark orange circle) B cells and their overlapping sub-populations.

(B) Proportions of the different sub-populations of CD11c^+^ B cells, relative to the number of CD11c^+^ B cells.

(C) Proportions of the different sub-populations of DN B cells, relative to the number of DN B cells.

(D) Proportions of the different sub-populations of CD21^-^CD27^-^ B cells, relative to the number of CD21^-^CD27^-^ B cells.

**Supplementary Figure 8: Overlap between CD11c^+^, DN and IL-10^+^ B cell populations**

(A) Reference Venn diagram showing the position of the 3 main populations CD11c^+^ (purple circle), DN (blue circle), and IL-10^+^ (yellow circle) B cells and their overlapping sub-populations.

(B) Proportions of the different sub-populations of CD11c^+^ B cells, relative to the number of CD11c^+^ B cells.

(C) Proportions of the different sub-populations of DN B cells, relative to the number of DN B cells.

(D) Proportions of the different sub-populations of IL-10^+^ B cells, relative to the number of IL-10^+^ B cells.

**Supplementary Figure 9: Gating strategies**

**(A)** Gating strategy used to identify cytokine producing B cells.

**(B)** Gating strategy used to identify CD21+ and CD73+ B cells.
